# The regenerative response of cardiac interstitial cells

**DOI:** 10.1093/jmcb/mjac059

**Published:** 2022-10-22

**Authors:** Laura Rolland, Alenca Harrington, Adèle Faucherre, Jourdano Mancilla Abaroa, Girisaran Gangatharan, Laurent Gamba, Dany Severac, Marine Pratlong, Thomas Moore-Morris, Chris Jopling

**Affiliations:** Institute of Functional Genomics, University of Montpellier, CNRS, INSERM, LabEx ICST, Montpellier34094, France; Institute of Functional Genomics, University of Montpellier, CNRS, INSERM, LabEx ICST, Montpellier34094, France; Institute of Functional Genomics, University of Montpellier, CNRS, INSERM, LabEx ICST, Montpellier34094, France; Institute of Functional Genomics, University of Montpellier, CNRS, INSERM, LabEx ICST, Montpellier34094, France; Institute of Functional Genomics, University of Montpellier, CNRS, INSERM, LabEx ICST, Montpellier34094, France; Institute of Functional Genomics, University of Montpellier, CNRS, INSERM, LabEx ICST, Montpellier34094, France; Institute of Functional Genomics, University of Montpellier, CNRS, INSERM, LabEx ICST, Montpellier34094, France; Montpellier GenomiX, France Génomique, Montpellier34094, France; Institute of Functional Genomics, University of Montpellier, CNRS, INSERM, LabEx ICST, Montpellier34094, France; Montpellier GenomiX, France Génomique, Montpellier34094, France; Institute of Functional Genomics, University of Montpellier, CNRS, INSERM, LabEx ICST, Montpellier34094, France; Institute of Functional Genomics, University of Montpellier, CNRS, INSERM, LabEx ICST, Montpellier34094, France

**Keywords:** heart regeneration, interstitial cells, single-cell RNA-sequencing, macrophage, fibroblast, endothelium, inflammation

## Abstract

Understanding how certain animals are capable of regenerating their hearts will provide much needed insights into how this process can be induced in humans in order to reverse the damage caused by myocardial infarction. Currently, it is becoming increasingly evident that cardiac interstitial cells play crucial roles during cardiac regeneration. To understand how interstitial cells behave during this process, we performed single-cell RNA sequencing of regenerating zebrafish hearts. Using a combination of immunohistochemistry, chemical inhibition, and novel transgenic animals, we were able to investigate the role of cell type-specific mechanisms during cardiac regeneration. This approach allowed us to identify a number of important regenerative processes within the interstitial cell populations. Here, we provide detailed insight into how interstitial cells behave during cardiac regeneration, which will serve to increase our understanding of how this process could eventually be induced in humans.

## Introduction

The very limited regenerative potential of the adult mammalian heart underlies an increasing prevalence of heart failure (Savarese and Lund, 2017). Studies using animal models such as the zebrafish and neonatal mice have shown that, following a substantial loss of myocardium, regeneration can be achieved through cardiomyocyte proliferation (Jopling et al., 2010; Porrello et al., 2011). Furthermore, it has become increasingly evident that this process cannot occur without a suitable environment that is provided by multiple interstitial cell populations. Notably, following injury, clearing of cellular debris, neovascularization, and extracellular matrix scaffold constitution require the highly coordinated activity of interstitial cells such as immune cells, endothelial cells, and fibroblasts (Fernandez et al., 2018; Forte et al., 2018).

Recent studies have harnessed the power of single-cell analysis to overcome difficulties associated with heterogeneous cell populations such as cardiac fibroblasts and macrophages. This has provided a more detailed overview of interstitial cell function after cardiac injury in adult mice and during cardiac regeneration in neonates (Farbehi et al., 2019; Wang et al., 2020). Although the regenerating neonatal mouse heart is highly relevant for identifying mechanisms that could help promote adult human heart regeneration, it is also actively remodeling when subjected to injury, meaning certain features relevant to achieving regeneration in the adult heart may be missing. The zebrafish represents a complementary model for exploring cardiac regeneration, as quiescent adult zebrafish myocardium is able to regenerate following significant injury (Jopling et al., 2010).

To further understand the process of cardiac regeneration in adult zebrafish, we performed single-cell analysis of interstitial cell populations in regenerating hearts. Furthermore, we provided a rigorous quantification of the different cell types present in the zebrafish ventricle, including cardiomyocytes, endothelium, epicardium, fibroblasts, macrophages, and erythrocytes. Among these cell types, our analysis revealed intriguing properties of fibroblasts, endothelial cells, and macrophages that support cardiac regeneration in adult zebrafish.

Fibroblasts are involved in multiple processes associated with the cardiac response to injury and have previously been shown to play a crucial role during cardiac regeneration in zebrafish (Sanchez-Iranzo et al., 2018). Our data indicates many similarities in the injury response between zebrafish cardiac fibroblasts and their adult mammalian counterparts; however, we have also identified significant differences, most notably a disparity in myofibroblast gene expression. Endothelial cells make up the bulk of the cardiac interstitial population. Recent studies have determined that endothelial neovascularization of the wound area is key for cardiac regeneration. This process lays down a framework, over which the regenerating myocardium can be formed (Fernandez et al., 2018). Here, we have determined that *tal1*, a gene essential for endocardial development, is required for cardiac regeneration in adult zebrafish. The macrophage response during cardiac regeneration also plays a pivotal role in ensuring a successful outcome (Aurora et al., 2014). For a long time, the balance between inflammation and regeneration has been regarded as one of the key elements of this process. Interestingly, our data indicate that the resident macrophages present in the uninjured adult zebrafish heart appear to display an inflammatory M1 signature, which, following injury, is rapidly eclipsed by M2-like macrophages. Furthermore, we have also determined that the matrix metalloproteinase (MMP), encoded by *mmp14*, is primarily expressed by M2-like macrophages and plays a crucial role in allowing them to invade the damaged tissue.

Our study underlines the importance and variety of interstitial cell functions that support adult zebrafish heart regeneration. Furthermore, we have also compared our findings with published single-cell RNA sequencing (scRNA-seq) studies of the interstitial cellular response in regenerating neonatal mice and non-regenerating adult mice following myocardial infarction (Farbehi et al., 2019; Wang et al., 2020). In doing so, we have endeavored to provide a balanced overview of the similarities and differences between regenerating and non-regenerating models.

## Results

### scRNA-seq of regenerating zebrafish ventricle

In order to analyze the regenerative response of different interstitial cell populations, we adopted a scRNA-seq strategy (Figure 1A). Following optimization of dissociation conditions and unbiased FACS-sorting of viable nucleated cells, we performed scRNA-seq (10× Chromium) of uninjured, sham-operated, and amputated (3, 7, and 14 days post-amputation (dpa)) adult zebrafish ventricles. Altogether, after quality control, we obtained 18739 transcriptional profiles and found that samples from uninjured and sham-operated zebrafish were largely comparable (Supplementary Figure S1A and Table S3). Unbiased clustering of cells from uninjured and amputated hearts revealed 15 clusters comprising 9 distinct cell types (Figure 1B and C; Supplementary Tables S1 and S2), whose proportions varied during the regenerative process (Figure 1B). Major cell types included *cdh5*^+^ endothelium, *mfap4^+^* myeloid cells (macrophages), *tcf21^+^* epicardium/epicardium-derived cells (EPDCs), *hbaa1^+^* erythrocytes, and *sla2^+^* lymphoid cells (Figure 1B and C). The latter included *lck^+^* T-lymphocytes and *pax5^+^* B-lymphocytes (Supplementary Figure S1B). Smaller populations included *mpx^+^* neutrophils, *itga2b^+^* thrombocytes, and *sox10^+^*cardiac neural crest (CNC) (and *rspo1^+^* neural crest derivatives) (Figure 1B and C). Although we could detect a small population of *myl7^+^* cardiomyocytes, these cells were largely absent from our dataset, presumably because their size was not compatible with our scRNA-seq pipeline (Figure 1B and C). Very rare ‘hybrids’, i.e. clusters expressing markers of multiple lineages, formed protrusions from cell type-specific clusters aimed in the direction of another cell type, as most obviously observed with the myocyte cluster (Figure 1B). Cell types were clearly separated in the UMAP plots, with an exception of the myeloid and lymphoid lineages. These populations included converging sub-populations characterized by a high expression of cell cycle-related genes (e.g. *mki67*) (Figure 1D–H; Supplementary Tables S1 and S2).

**Figure 1:**
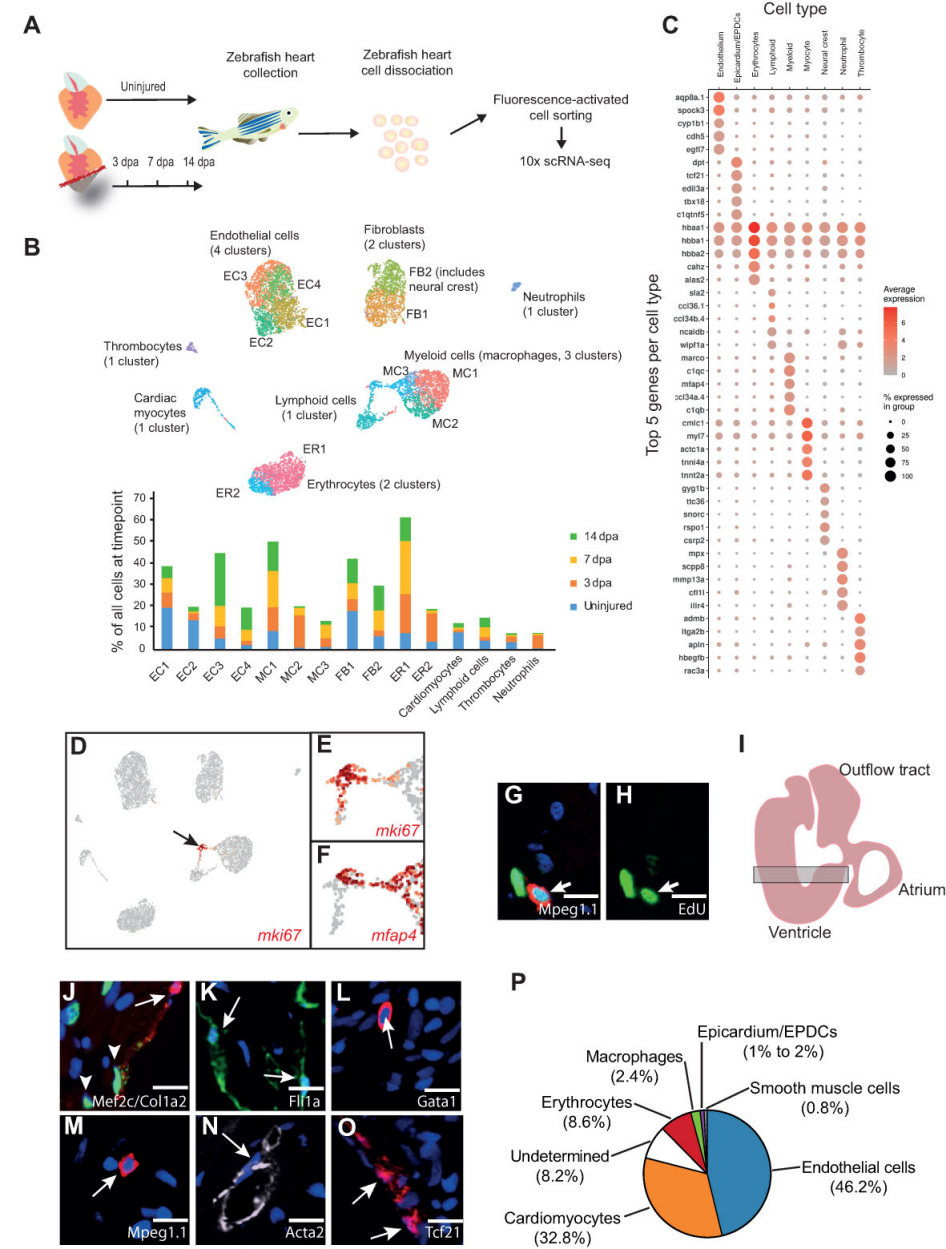
scRNA-seq analysis of regenerating zebrafish hearts. (**A**) Schematic of our scRNA-seq pipeline. (**B**) UMAP clusters of the different populations of cells and sub-clusters identified in zebrafish hearts. The graph shows the proportion of all the cells within individual clusters at each timepoint, e.g. EC1 contains 20% of all the cells in an uninjured ventricle, which drops to ∼5% at 3 dpa. ER, erythrocytes. (**C**) A DotPlot shows the 5 genes used to characterize the different cell types. (**D**–**F**) UMAP plot indicates the cluster of proliferating cells based on the expression of *mki67*. (**E**) The cluster pointed by the black arrow is shown at higher magnification. (**F**) The proliferating cluster shown contains *mfap4^+^* myeloid cells (macrophages) and *mfap4^–^*lymphoid cells.

**Figure 1:**
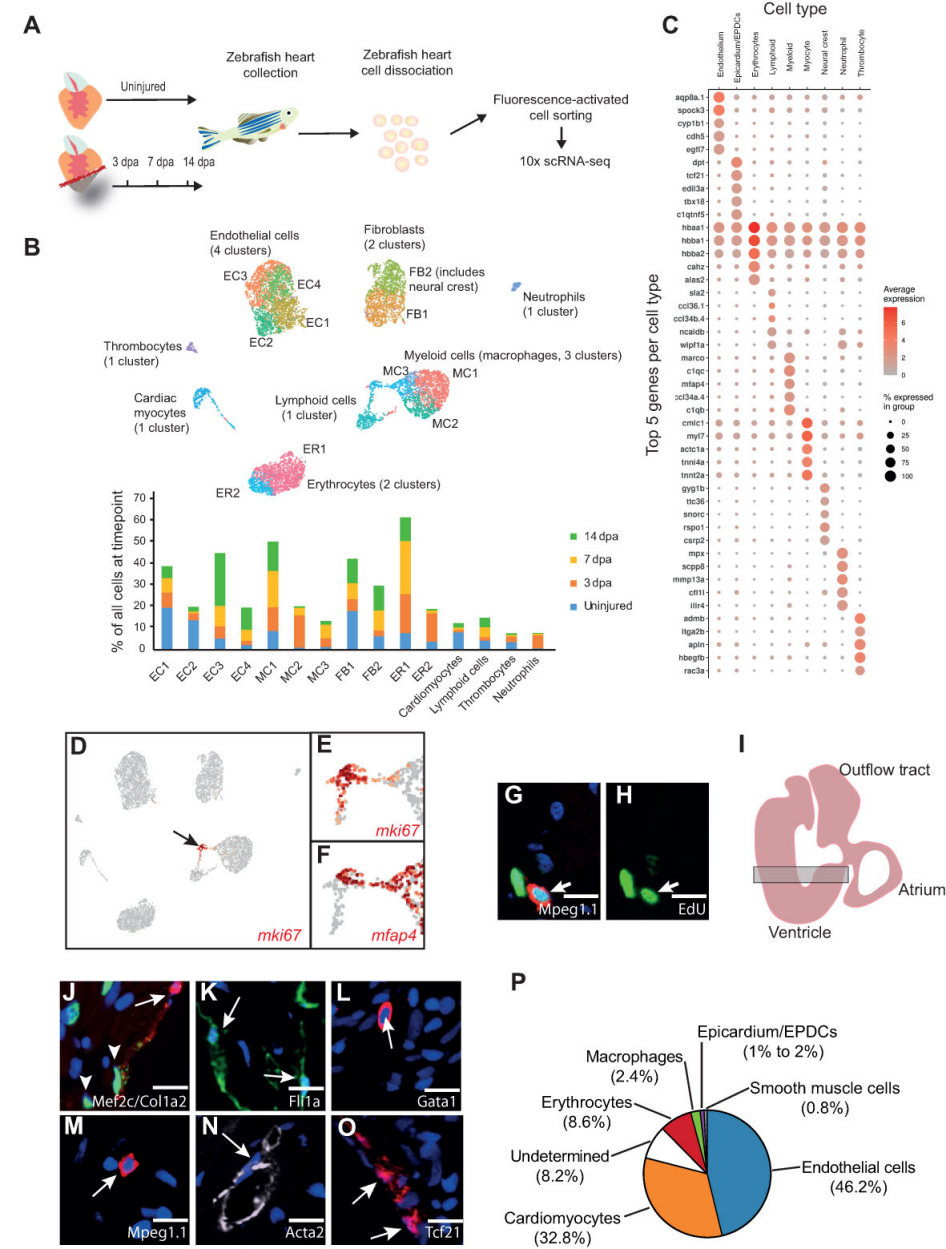
*(Continued)* (**G** and **H**) IHC of EdU-labelled 3 dpa regenerating zebrafish hearts. Mpeg1.1 (red) labels macrophages, EdU (green) labels proliferating cells, and DAPI (blue) labels nuclei. Scale bar, 10 μm. (**I**) Diagram of an adult zebrafish heart. The shaded rectangle indicates the area used to count the different cell types. (**J**–**O**) Examples of IHC images used to count the different cell types. Mef2c (green) labels cardiomyocytes, Col1a2:mCherry (red) labels fibroblasts, Fli1a:GFP (green) labels endothelium, Gata1:RFP (red) labels erythrocytes, Mpeg1.1:mCherry (red) labels macrophages, Acta2 (white) labels smooth muscle cells, and Tcf21:DsRed2 (red) labels fibroblasts. Scale bar, 10 μm. (**P**) Pie chart represents the proportions of different cell types present within the uninjured adult zebrafish ventricle. The average percentage of cells positive for each cell type-specific marker were identified among at least 800 cells per heart, with 5 hearts analyzed for each marker.

Loss of large and/or fragile cells, most notably cardiomyocytes and endothelial cells, can lead to under-representation of these cell types in scRNA-seq datasets (Farbehi et al., 2019). Accurate evaluation of the relative proportions of these cell types, in particular at baseline, is essential for contextualizing cell type-specific responses to injury. Previous studies have established that the adult zebrafish heart is mainly composed of cardiomyocytes and endothelium (Patra et al., 2017), but the proportions of other key cell types, including resident macrophages and epicardium/EPDCs, have not previously been described. Using immunohistochemistry (IHC) and several zebrafish transgenic lines as described in Materials and methods, we were able to assign a cell-type identity to 92.8% of DAPI^+^ nuclei in the ventricle (Figure 1I–P). These included Mef2c^+^ cardiomyocytes (32.8%), Fli1a:GFP*^+^* endothelial cells (46.2%), Gata1:DsRed*^+^* erythrocytes (8.6%), Mpeg1.1:mCherry*^+^* macrophages (2.4%), Col1a2:mCherry^+^ and Tcf21:DsRed*^+^*epicardium/EPDCs (1%–2%), and Acta2^+^ smooth muscle cells (0.8%) (Figure 1J–P). It was essential to clearly identify and quantify zebrafish erythrocytes, as they are nucleated in this species. We were unable to assign an identity to 8.2% of nuclei that may include cells with a weak reporter/IHC signal and rare populations such as lymphocytes and CNC-derived cells. Hence, as in the adult mouse heart, the endothelial and cardiomyocyte populations represented the most abundant cell types (Pinto et al., 2016). However, the uninjured adult zebrafish heart presented a relatively low number of epicardium/EPDCs such as fibroblasts. Indeed, we evaluated that epicardium/EPDCs represented 1%–2% of the cells in the adult zebrafish heart. In comparison, 11% of the adult mouse heart is comprised of fibroblasts (Pinto et al., 2016).

#### Macrophages

Our analysis revealed three macrophage clusters (MCs), of which MC1 was predominant in uninjured hearts. The top gene associated with the resident MC1 vs. other MCs was *cxcr3.3*, a ligand scavenging receptor associated with reduced macrophage mobility (Sommer et al., 2020). These cells also expressed the highest levels of markers of activated M1 macrophages (*tnfa, il1b, cd40*, and *il6r*) and neutrophil-recruiting chemokines (*cxcl8a* and *csf3b*) (Figure 1B; Supplementary Figure S1C and Table S1). MC2 was predominant at 3 dpa and expressed high levels of genes associated with M2-like properties, including *ctsc* and *c1qa* (Figure 1B; Supplementary Figure S1C and Table S1). Also, MC2 expressed genes associated with recruited macrophages, such as *ccr2* (Epelman et al., 2014) and *apoeb* (Mould et al., 2019), as well as tissue healing, including the copper chaperone *atox1* (Das et al., 2016; Supplementary Figure S1C and Table S1). MC3 was characterized by a very strong cell cycle-related gene signature, including *mki67, top2a, pcna*, and *cdk1* (Figure 1D–F; Supplementary Table S1). In support of this, we were able to directly observe proliferating EdU-labelled macrophages in regenerating ventricles by IHC analysis (Figure 1G and H).

#### Endothelium

Based on cell counts, we determined that 46.2% of the cells in the ventricle were endothelial/endocardial. Interestingly, our scRNA-seq data clearly showed that, at both baseline and following resection, endocardial endothelium had both endothelial (*cdh5*) and mesenchymal (*pdgfr*α and *col1a2*) signatures, as previously observed in the mouse (Moore-Morris et al., 2014). We obtained four endothelial clusters (ECs), all showing strong expression of pan-endothelial markers *aqp8a.1, cdh5*, and *vwf* (Supplementary Table S1). EC1 and EC2 were abundant in control hearts. EC1 was characterized by expression of relatively high levels of collagen (*col1a1a, col1a1b*, and *col1a2*) (Figure 1B; Supplementary Figure S1D and Table S1). Also, EC1 was the most proliferative EC, with 1.9% *mki67^+^* cells at 7 dpa (Supplementary Table S1). EC2 was characterized notably by *nppc*, a key regulator of vascular homeostasis (Moyes et al., 2014), whose receptor *npr3* was expressed by EPDCs at baseline (Supplementary Figure S1D and Table S1). EC3 and EC4 increased in size over time following amputation (Figure 1B). EC3 expressed relatively high levels of several heme-binding genes (*hbba1, hbba2, hbaa1*, and *hbaa2*), albeit at a far lower level than erythrocytes (Supplementary Figure S1D and Tables S1 and S2). Markers of venous endothelium, such as *kdrl*, were not specific to any EC (Supplementary Figure S1D). Markers of lymphatic endothelium *prox1a* and *lyve1b* were expressed by a small subset of endothelial cells that did not segregate to any specific cluster (Supplementary Figure S1D and Table S1).

#### Epicardium/EPDCs

Within our dataset, we could clearly delineate two epicardial/EPDC clusters, which are the major source of fibroblasts (FB). FB1 was characterized by a high expression of genes associated with extracellular matrix organization (*adamstl2* and *col18a1b*) and integrin binding (*edil3a* and *hapln1a*) (Supplementary Figure S1E and Table S1). FB1 was the most proliferative epicardium/EPDC cluster, with a peak of proliferation at 3 dpa (3.6% *mki67^+^*) (Supplementary Table S1). Cells in FB2 were notably characterized by a high expression of genes involved in complement activation (*c4, c4b*, and *c6*) (Supplementary Figure S1E and Table S1). Proportionately, FB1 was most abundant in unamputated hearts, whereas cell numbers in FB2 increased following injury (Figure 1B). We were able to clearly identify epicardium and epicardial-derived fibroblasts based on their expression of the previously described fibroblast-specific gene *tcf21* (Tallquist and Molkentin, 2017; Figure 1C; Supplementary Tables S1 and S2). Clustering did not seem to reflect epicardial- vs. epicardium-derived cells. For example, *aldh1a2^+^* and *clu^+^* epicardial cells were present in subsets of both FB1 and FB2 (Supplementary Figure S1E and Table S1).

#### Neural crest

We were also able to identify a *sox10^+^* population of CNC-derived cells, which were assigned to the FB2 cluster, probably because of relatively low number of cells (Figures 1B and 2A). We classified these cells as CNC-derived based on the high expression of markers of neural crest cells (*sox10^+^, pax3a^+^*, and *apoda.1^+^*) and CNC-derived mesenchyme (*rspo1^+^, gyg1b^+^, csrp2^+^*, and *hand2*^high^) (Figure 2A; Supplementary Tables S1 and S2). Strikingly, these cells did not express the epicardium/EPDC-specific genes (*tcf21* and *tbx18*) (Figure 2A) nor the FB2-specific gene *adh8a* (Supplementary Table S1). Within the CNC cluster, we could clearly distinguish *sox10*^high^;*rspo1^–^* and *sox10*^low^;*rspo1^+^* sub-clusters. Previous studies in zebrafish have shown that *sox10*-expressing cells are concentrated in the atrioventricular valves (Sande-Melon et al., 2019). In mice, *Rspo1* has recently been reported to be expressed in epicardial cells (Wang et al., 2020). IHC analysis of neonatal mouse hearts confirmed that Rspo1 was indeed associated with the epicardial layer (Supplementary Figure S2A). Similarly, IHC revealed that Rspo1^+^ cells were tightly associated to Tcf21:DsRed2*^+^*epicardial cells (Figure 2B–D). However, re-analysis of the scRNA-seq data by Wang et al. (2020) confirmed that, in contrast to what we observed in zebrafish, murine *Rspo1^+^* cells were clearly expressing epicardial/EPDC markers such as *Tcf21* and *Tbx18* (Supplementary Figure S2B), underlining a divergence in the profiles of *Rspo1^+^* cells in these species.

**Figure 2:**
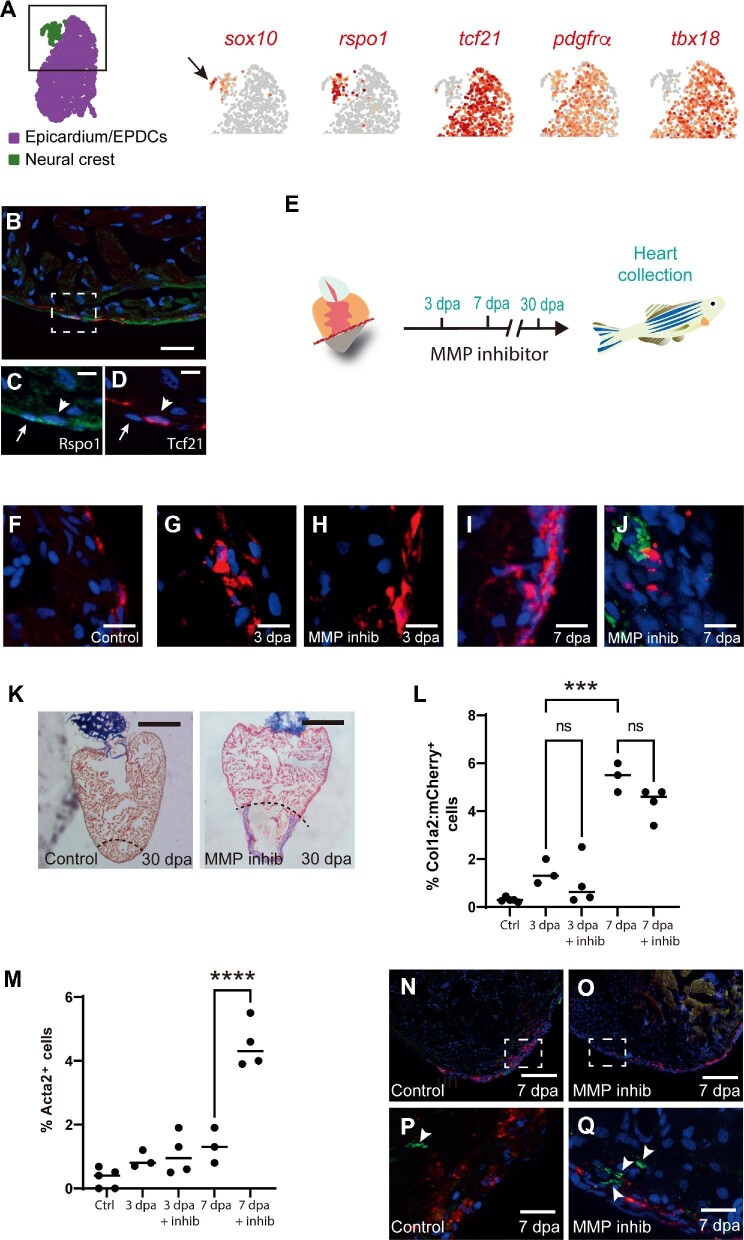
Mesenchymal lineages and cardiac fibroblast activation in the regenerating zebrafish heart. (**A**) UMAP plot indicates the epicardial (purple) and neural crest (green) clusters. The black square delineates the area depicted in the subsequent UMAP plots. UMAP plots depict the relative expression of *sox10* (neural crest; the black arrow indicates the neural crest cluster), *rspo1, tcf21* (epicardium/EPDCs),

**Figure 2:**
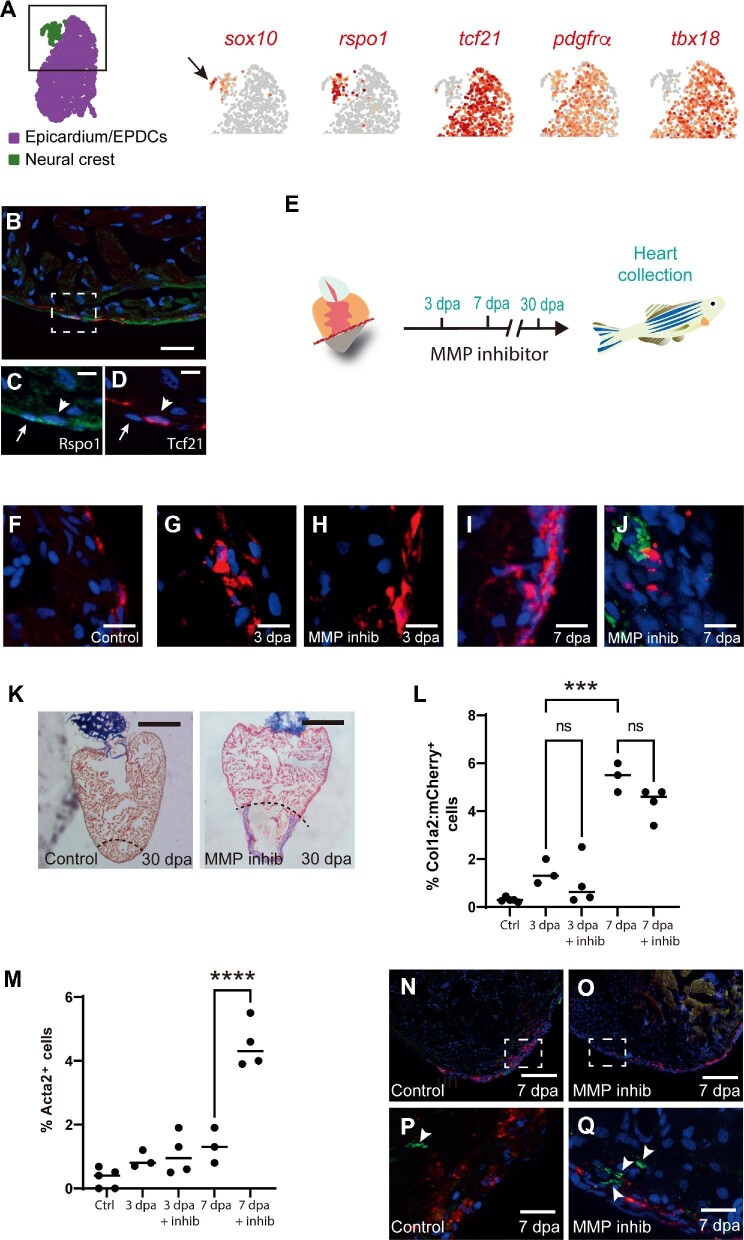
*(Continued) pdgfr*α (epicardium/EPDCs), and *tbx18* (epicardium/EPDCs). Note that *rspo1* is restricted to the neural crest cluster and does not segregate with either *tcf21* or *tbx18*. (**B**) An IHC image of a *Tg(tcf21:dsRed2)* zebrafish heart stained for Rspo1 (green) and Tcf21:DsRed2 (red). Scale bar, 20 μm. (**C** and **D**) Higher magnification images of the region highlighted in **B** show Rspo1 (green) and Tcf21:DsRed2 (red). The white arrow indicates a Rspo1-expressing cell and the white arrow head indicates a Tcf21-expressing cell. Scale bar, 5 μm. (**E**) Schematic of MMP inhibitor (GM6001) treatment protocol. (**F**–**J**) IHC images of *Tg(col1a2:mCherry)* zebrafish hearts show Col1a2:mCherry (red) and Acta2 (green) in an uninjured control heart (**F**), a 3 dpa heart (**G**), a 3 dpa MMP inhibitor (GM6001)-treated heart (**H**), a 7 dpa heart (**I**), and a 7 dpa MMP inhibitor (GM6001)-treated heart (**J**). Scale bar, 10 μm. Note the absence of Col1a2:mCherry and Acta2 co-localization. (**K**) AFOG staining of control and MMP inhibitor (GM6001)-treated zebrafish hearts at 30 dpa. The black dashed line indicates the plane of amputation. Note the presence of a large fibrin (red)/collagen(blue) scar. Scale bar, 400 μm. (**L**) The graph shows the percentage of cells, from an average of 650 cells per heart, that were Col1a2:mCherry^+^ in the different experimental conditions. (**M**) The graph shows the percentage of cells, from an average of 650 cells per heart, that expressed Acta2 (non-vascular) in the different experimental conditions. Note that inhibitor treatment significantly increases the number of Acta2^+^ cells. (**N** and **O**) Low magnification IHC images of *Tg(col1a2:mCherry)* zebrafish hearts show Col1a2:mCherry (red) and Acta2 (green) at 7 dpa with or without MMP inhibitor (GM6001) treatment. Scale bar, 100 μm. (**P** and **Q**) Higher magnification images of the regions highlighted in **N** and **O**, respectively. White arrow heads indicate Acta2-expressing cells (green). Scale bar, 20 μm. *P*-values were calculated by one-way ANOVA. ***P < 0.001, ****P < 0.0001; ns, non-significant.

### A hallmark of activated mammalian cardiac fibroblast is absent in zebrafish

Following injury, we observed a strong fibrotic response, which was initiated in epicardium/EPDCs, notably with a robust upregulation of *periostin* (*postnb*) and *fibronectin* (*fn1a/b*) expression (Humeres and Frangogiannis, 2019; Supplementary Figure S2C and Tables S1 and S2). In contrast, *acta2*, a gene associated with injury-induced myofibroblast activation in mammals (Humeres and Frangogiannis, 2019), was not upregulated at any timepoint in the epicardium/EPDC lineage (Supplementary Figure S2C). To confirm this observation, we performed cardiac amputations on *Tg(col1a2:loxP-mCherry-NTR)* zebrafish followed by IHC for Acta2 at 3 dpa and 7 dpa (Figure 2E–Q). Surprisingly, although the number of Col1a2:mCherry^+^ cells increased following injury, this was not accompanied by an increase in Acta2-expressing cells (Figure 2L and M). Elevated Acta2 production can be observed in non-regenerating adult mammalian hearts following injury and, similarly, an increase in Acta2 production has also been observed in adult zebrafish mutants, which are unable to regenerate their hearts (Xu et al., 2019). Based on these observations, we surmised that inhibiting cardiac regeneration in adult zebrafish may also lead to an increase in Acta2 production and allow us to further investigate whether or not this was associated with fibroblasts. The pan-MMP inhibitor GM6001 has previously been reported to significantly inhibit zebrafish cardiac regeneration (Lien et al., 2006). In agreement with this, we also found that treating adult zebrafish with GM6001 following apical resection resulted in a failure to regenerate at 30 dpa and the formation of a large fibrin/collagen scar (Figure 2K). Using this protocol, we performed cardiac amputations on *Tg(col1a2:loxP-mCherry-NTR)* zebrafish followed by IHC for Acta2 in the lower ventricle at 3 dpa and 7 dpa (Figure 2H and J). Interestingly, inhibiting cardiac regeneration with GM6001 led to a significant increase in the number of interstitial Acta2^+^ cells at 7 dpa (Figure 2M). Furthermore, Acta2 did not co-localize with Col1a2:mCherry, indicating that cell types other than fibroblasts were upregulating Acta2 expression (Figure 2N–Q). This is in agreement with a recent study that reported the expression of smooth muscle-specific genes outside the epicardial lineage, notably in the endocardium (Koth et al., 2020). However, because GM6001 lacks specificity, we also acknowledge that further research will be required using multiple zebrafish models in order to establish these findings conclusively. To determine whether the expression of *Acta2* by fibroblasts varies between species, we re-analyzed two previously published scRNA-seq datasets from non-regenerating adult mice and regenerating neonatal mice after myocardial infarction (Farbehi et al., 2019; Wang et al., 2020; Supplementary Figure S2C). Interestingly, the sustained expression of the fibrosis-associated gene, *Postn*, 3 days after injury appeared remarkably similar between adult mice and zebrafish (Supplementary Figure S2C). On the other hand, following myocardial infarction in adult mice and postnatal day 8 (P8) neonates, there was a robust upregulation of *Acta2* expression by fibroblasts 3 days post injury, a feature which was absent in the adult zebrafish after cardiac injury (Supplementary Figure S2C). The picture in regenerating P1 neonate fibroblasts was far less clear, as they already expressed *Acta2* at baseline along with high levels of *Postn*, presumably because the P1 neonatal heart is still undergoing widespread remodeling (Supplementary Figure S2C). Taken together, our data indicate that collagen-producing cardiac fibroblasts in adult zebrafish do not upregulate Acta2 in response to injury.

### Tal1 is a regulator of the endothelial regenerative response

The zebrafish endothelial response to injury involves a rapid change in gene expression followed by wound neovascularization, a process which lays the foundation for the proliferating cardiomyocytes to regenerate the missing myocardium (Fernandez et al., 2018). A number of genes have been directly linked to the endothelial regenerative response in zebrafish such as *vegfaa, aldh1a2*, and *notch1b* (Kikuchi et al., 2011; Marin-Juez et al., 2016; Munch et al., 2017). We could not detect any significant upregulation of any of these genes, which could be due to rapid changes in their expression occurring outside the timepoints analyzed here, as reported for *vegfaa*. On the other hand, although the average level of expression of *notch1b* in the endothelium did not change significantly, we detected an increase (8%) in the proportion of endothelial cells expressing this gene at 3 dpa compared with uninjured controls. The re-expression of developmental genes is also a hallmark of regenerating endothelium (Fernandez et al., 2018). We found that the proportion of cells expressing genes required for endothelial development (e.g. *foxc1a* and *foxc1b*) increased during cardiac regeneration (Supplementary Figure S3A and B; De Val, 2011). The BHLH transcription factor Tal1 is also essential for endocardial development and for maintaining endocardial identity, in particular for establishing endothelial Tjp1 tight junctions (De Val, 2011). Our scRNA-seq indicates that Tal1 is primarily expressed in endothelial cells, erythrocytes, and thrombocytes (Supplementary Figure S3C). Previous research has shown that *tal1* is upregulated during cardiac regeneration in zebrafish (Jopling et al., 2012). In agreement with this, our scRNA-seq analysis indicates that there was an increase in the proportion of *tal1*-expressing endothelial cells during regeneration (Figure 3A and B). This increase peaked at 7 dpa before returning to pre-injury levels by 14 dpa (Figure 3B). Without detailed lineage tracing, we are presently unable to determine whether the increase in *tal1*-expressing endothelial cells was due to the proliferation of existing *tal1*-expressing cells or because more endothelial cells have begun producing *tal1 de novo*. To confirm our scRNA-seq data, we performed IHC for Tal1 on adult *Tg(fli1a:GFP)y1* zebrafish cardiac sections and were able to clearly observe Tal1*^+^* endothelial cells (Figure 3C–E). Because Tal1 is an obligate dimer and can form complexes with a variety of proteins, which will subsequently dictate which transcriptional programs to activate/deactivate (Stanulovic et al., 2017), we extended our analysis to known co-factors of Tal1. LIM only 2 (LMO2) forms a multi-protein complex with Tal1 and directs it towards specific target genes. Previous research indicates that in the absence of LMO2, Tal1 is able to target other genes for expression/repression (Stanulovic et al., 2017). We found that *lmo2* was downregulated during the early stages of regeneration (Supplementary Figure S3D). In order to analyze this in more detail, we re-clustered *tal1^+^* endothelial cells to identify changes in gene expression associated with this sub-population (Supplementary Figure S4A). Interestingly, this analysis revealed that although *lmo2* expression was evenly distributed in three of the *tal1^+^* clusters (T1, T2, and T3), it was reduced in the fourth cluster (T4) (Supplementary Figure S4B). Furthermore, the expression of the *tal1* target gene *cgnl1*, a component of tight junctions and implicated in neovascularization, shows the highest level of expression in cluster T4 (Supplementary Figure S4C). We could also observe a downregulation of *lmo2* at 3 dpa and 7 dpa specifically in *tal1^+^* endothelial cells (Supplementary Figure S4D). Conversely, *cgnl1* became upregulated in *tal1^+^* cells at 7 dpa (Supplementary Figure S4E). Although these observations were below the threshold of significance (*P* < 0.05), this evident trend prompted us to target *tal1* directly to determine whether this core endocardial developmental gene could be involved in cardiac regeneration. In order to achieve this, we generated a transgenic zebrafish line, *fliEP:Ert2CreErt2;fliEP:loxRFPlox:DNtal1*, which can express a dominant negative (DN) Tal1 isoform specifically in endothelial cells following treatment with tamoxifen (4OHT) (Supplementary Figure S4F–P). The DN Tal1 isoform lacks the basic DNA-binding domain but is still able to form multi-protein transcription complexes and thus inhibits native Tal1-associated transcription (Aplan et al., 1992). We first assessed whether this transgenic line was functional by inducing DN *tal1* expression during early zebrafish development. This procedure caused cardiac developmental defects reminiscent of those previously described for *tal1* knockout zebrafish (Supplementary Figure S4N–P). To determine whether *tal1* is required for heart regeneration, we induced expression of DN *tal1* prior to cardiac resection (Figure 3F). Histological staining of heart sections at 30 dpa revealed that expression of DN *tal1* inhibited the regenerative process, as evidenced by a large fibrin/collagen scar (Figure 3G–K). To gain further insight into how regeneration had been disrupted, we labelled proliferating cardiomyocytes with EdU and Mef2c. In this manner, we were able to determine that expressing DN *tal1* significantly inhibited cardiomyocyte proliferation (Figure 3L–N). Revascularization of the wound is a critical process during regeneration and could potentially be disrupted when Tal1 signalling is blocked. To assess this possibility, we analyzed the formation of the vascular plexus at 7 dpa. In control hearts, we could clearly delineate the regenerating vascular plexus; however, this feature was significantly reduced in regenerating hearts expressing DN *tal1* in their endothelium (Figure 3O–S). Lastly, to determine whether the expression of *tal1* in the endothelium is zebrafish-specific, we re-analyzed two previously published scRNA-seq datasets from non-regenerating adult mice and regenerating neonatal mice after myocardial infarction and observed that *Tal1* is present within the endothelial populations (Farbehi et al., 2019; Wang et al., 2020; Supplementary Figure S5). Taken together, these data indicate that *tal1* is a key regulator of the endothelial response during cardiac regeneration.

**Figure 3:**
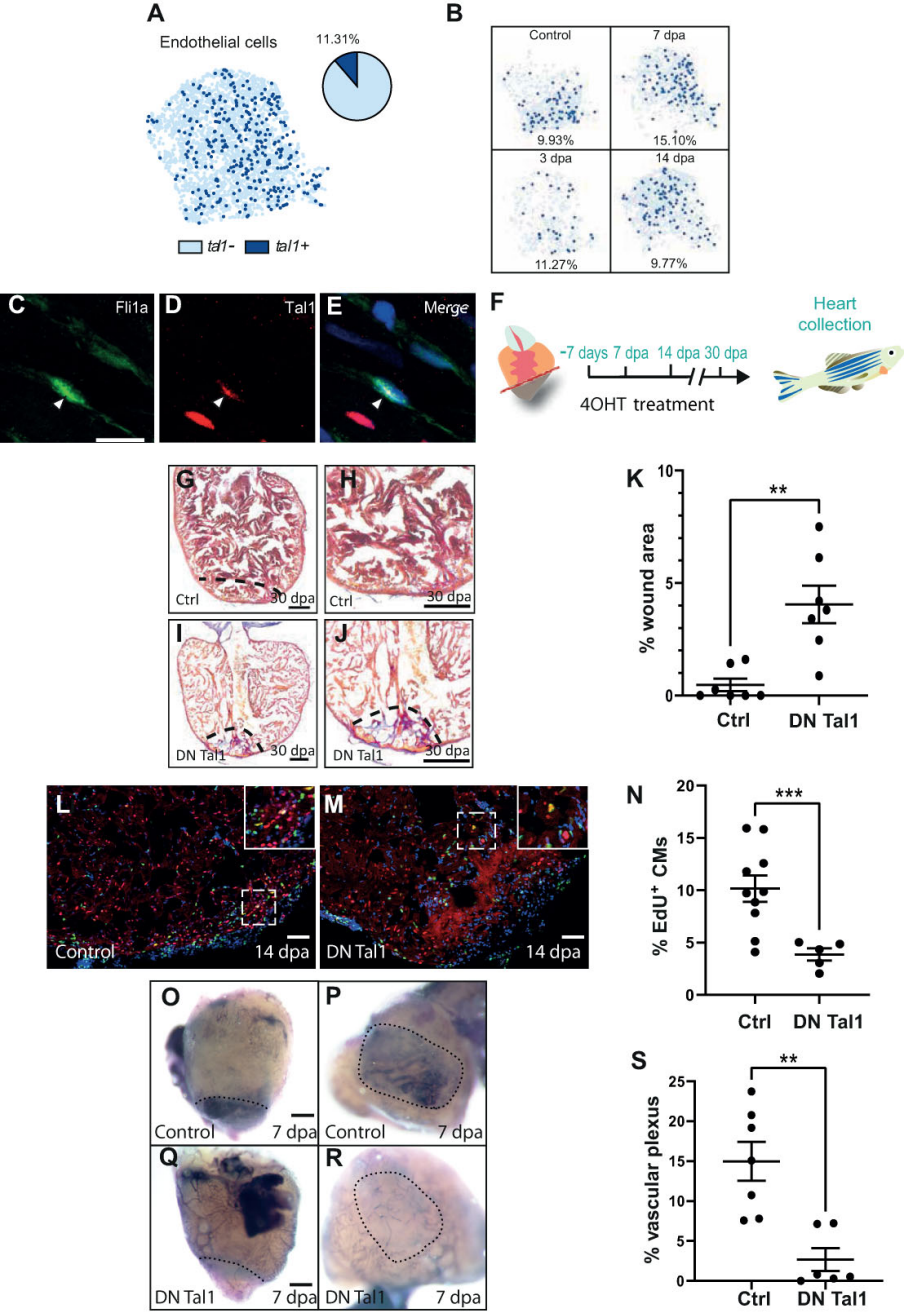
Tal1 is a regulator of the endothelial regenerative response. (**A**) UMAP plot of the endothelial cell cluster (light blue dots) shows that *tal1* is expressed in a subset of these cells (dark blue dots). The pie chart indicates the total proportion of endothelial cells that express *tal1*. (**B**) UMAP plots of the endothelial population at different stages of regeneration (control/unamputated, 3 dpa, 7 dpa, and 14 dpa). The proportion of *tal1*-expressing endothelial cells (dark blue dots) is given as a percentage underneath each UMAP plot. (**C**–**E**) IHC analysis of endothelial cells. Fli1a*^+^*, green (**C**); Tal1*^+^*, red (**D**); a merged image (**E**). Scale bar, 10 μm. (**F**) Schematic of the 4OHT treatment.

**Figure 3:**
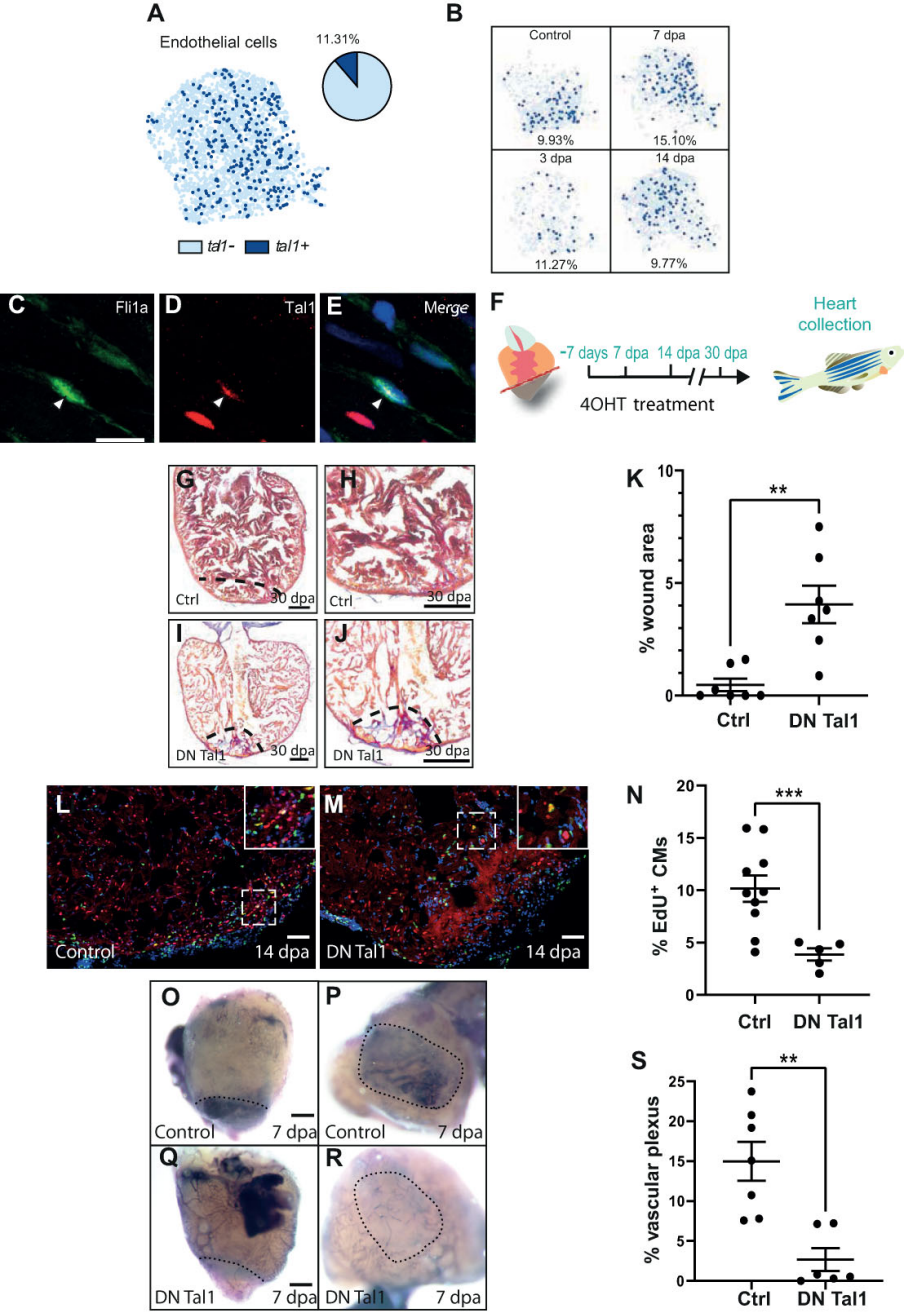
*(Continued)* (**G**–**J**) AFOG staining of a control (**G** and **H**) or DN Tal1-expressing (**I** and **J**) heart at 30 dpa. The black dashed line indicates the plane of amputation. The same images are shown in **H** and **J** at higher magnification, respectively. Note the presence of a large fibrin (red)/collagen (blue) scar. Scale bar, 200 μm. (**K**) The graph indicates the average area of the wound region as a percentage of the entire ventricle (control *n* = 7, DN Tal1 *n* = 7). (**L** and **M**) EdU labelling of cycling cardiomyocytes in a control (**L**) or DN Tal1-expressing (**M**) heart. MEF2/cardiomyocytes (red), EdU (green). The dashed white box indicates the area shown at higher magnification in the inset. Scale bar, 100 μm. (**N**) The graph indicates the percentage of EdU^+^ cardiomyocytes in the entire ventricle (control *n* = 10, DN Tal1 *n* = 5). CMs, cardiomyocytes. (**O**–**R**) Alkaline phosphatase staining of the regenerating vascular plexus in a control (**O** and **P**) or DN Tal1-expressing (**Q** and **R**) heart. The same hearts viewed dorsally at higher magnification are shown in **P** and **R**, respectively. The black dashed line indicates the wound/myocardium border (**O** and **Q**) or wound region (**P** and **R**). Scale bar, 200 μm. The reduced alkaline phosphatase staining indicates that the vascular plexus has failed to regenerate normally. (**S**) The graph indicates the average area of the vascular plexus as a percentage of the entire wound region (control *n* = 7, DN Tal1 *n* = 6). *P*-values were calculated using a non-parametric Mann–Whitney test. *P < 0.1, **P < 0.01, ***P < 0.001, ****P < 0.0001; DN, dominant negative.

### MMP14-expressing macrophages are required for cardiac regeneration

Macrophages play a crucial role during cardiac regeneration (Aurora et al., 2014). We were able to identify three clusters of macrophages within our scRNA-seq dataset. As described earlier, MC1 likely represents a resident population of inflammatory/M1 macrophages, which was predominant in uninjured hearts (Figures 1B and 4A). MC2 macrophages appeared at 3 dpa, persisted through 7 dpa, and resolved by 14 dpa (Figures 1B and 4A). This cluster was enriched for transcripts commonly associated with M2-polarised macrophages such as *cd9b* and inflammasome-associated genes (*txnipa, caspa*, and *atp13a2*) (Chitu and Stanley, 2006; Franchi et al., 2009; Qiao et al., 2016; Brosseau et al., 2018; Supplementary Figure S6A–D and Table S1). Furthermore, MC2 was also enriched for genes previously described to be associated with regenerating macrophages in adult zebrafish (*fabp11a, lgals9l1*, and *lgmn*) (Mitchell et al., 2019; Supplementary Figure S6E–G and Table S1). Lastly, MC3 was enriched for genes associated with the cell cycle, such as *pcna* and *mki67*, indicating that macrophage proliferation also occurs during cardiac regeneration in zebrafish, similar to the proliferation observed in mouse hearts following myocardial infarction (Sager et al., 2016; Supplementary Figure S7A, B and Table S1).

**Figure 4:**
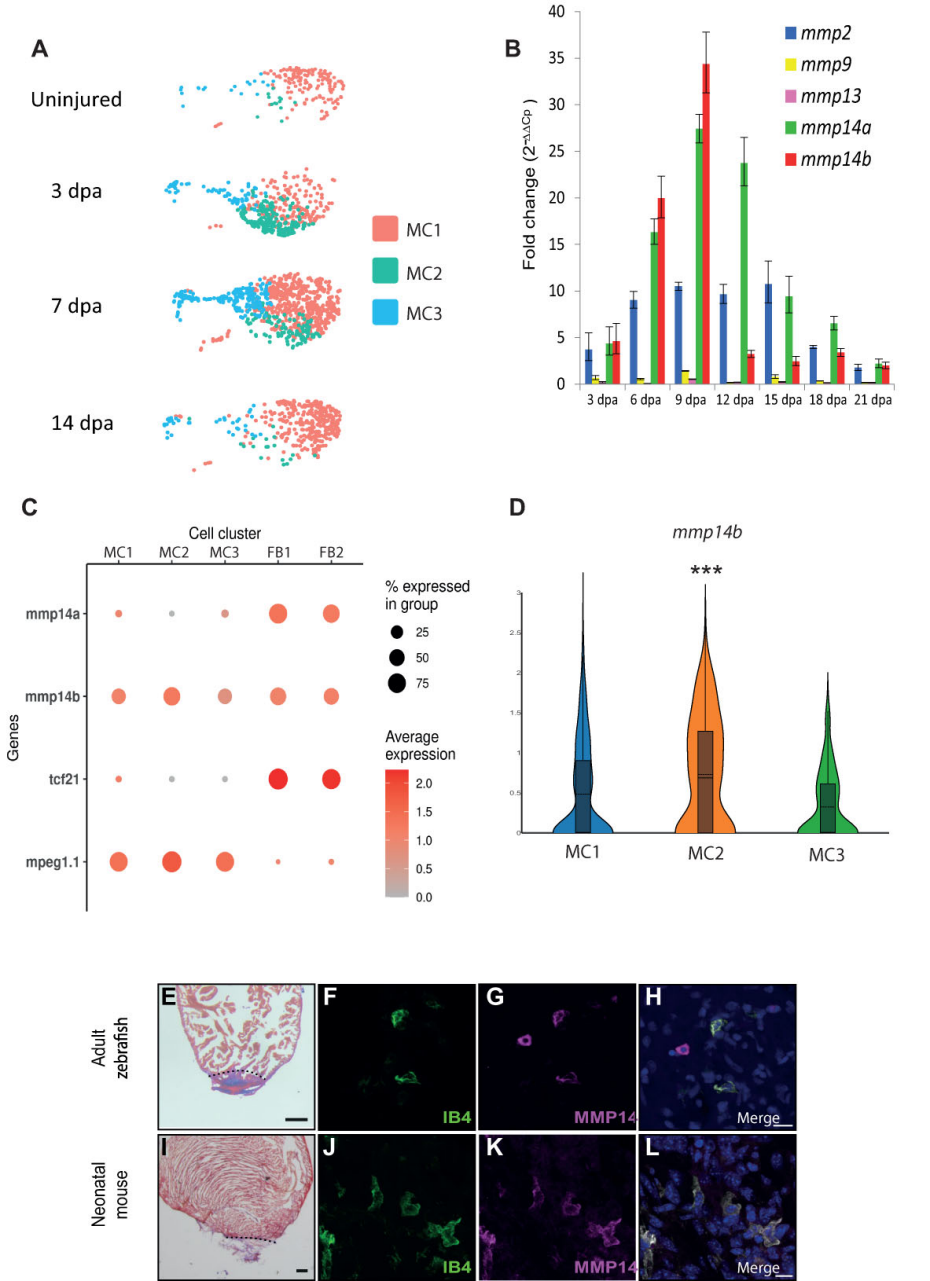
MMP14 is expressed by macrophages during cardiac regeneration. (**A**) UMAP plots of the macrophage population at different stages of regeneration (uninjured, 3 dpa, 7 dpa, and 14 dpa). The colored boxes indicate the different MCs (MC1, MC2, and MC3). (**B**) RT–qPCR analysis of *mmp* expression during cardiac regeneration. (**C**) A DotPlot shows the relative expression levels of *mmp14a* and *mmp14b* in macrophages (MC1, MC2, and MC3) and fibroblasts/epicardium (FB1 and FB2). *tcf21* was used to identify the fibroblast population and *mpeg1.1* was used to identify the macrophage population. (**D**) Violin plot compares the expression of *mmp14b* in MC1, MC2, and MC3.

**Figure 4:**
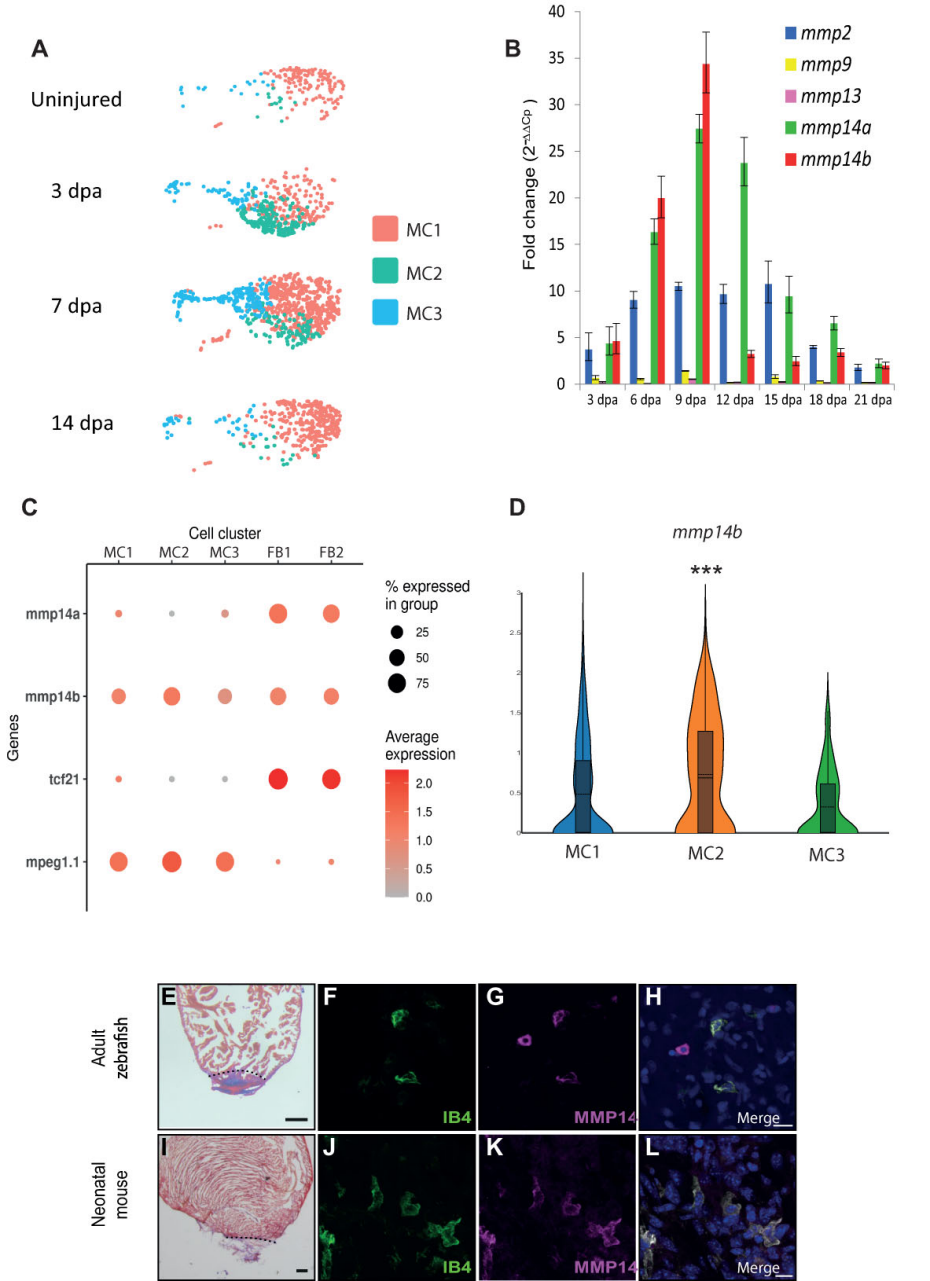
*(Continued)* (**E**) AFOG staining of a wild-type zebrafish heart at 7 dpa. The black dashed line indicates the plane of amputation. Note the presence of a large fibrin (red)/collagen (blue) scar. Scale bar, 200 μm. (**F**–**H**) IHC of a 7 dpa zebrafish heart section for IB4 (green, macrophages) (**F**), MMP14 (magenta) (**G**), and a merged image (**H**). Scale bar, 10 μm. (**I**) AFOG staining of a neonatal mouse heart section at 7 dpa. The black dashed line indicates the plane of amputation. Note the presence of a large fibrin (red)/collagen (blue) scar. Scale bar, 200 μm. (**J**–**L**) IHC on a neonatal mouse heart section at 7 dpa for IB4 (green, macrophages) (**J**), MMP14 (magenta) (**K**), and a merged image (**L**). Scale bar, 10 μm. *P*-values were adjusted using the Benjamini–Hochberg correction. *P < 0.1, **P < 0.05, ***P < 0.01, ****P < 0.001.

The expression profiles of *tnfa* and *il1b* are commonly used to distinguish between M1 and M2 macrophage polarization. We observed that the highest expression level of *tnfa* was in uninjured resident macrophages (Supplementary Figure S8A). In agreement with this, we also observed that the expression of *txnipa*, a gene whose expression is negatively regulated by *tnfa* (Levring et al., 2019), was sharply upregulated at 3 dpa as *tnfa* expression subsided (Supplementary Figure S8B). Furthermore, resident macrophages also expressed higher levels of *il1b* compared to the MC2 population (Supplementary Figure S1C). Because this appears to be at odds with previous reports, which indicate that resident macrophages do not usually display a pro-inflammatory signature (Swirski and Nahrendorf, 2013; Ma et al., 2018), we re-analysed two previously published scRNA-seq datasets from non-regenerating adult mice and regenerating neonatal mice after myocardial infarction (Supplementary Figure S9). Expression of the chemokine receptor Ccr2 has previously been shown to identify recruited macrophages following myocardial injury (Epelman et al., 2014). During cardiac regeneration in P1 neonatal mice, there was an increase (albeit non-significant) in macrophage *Ccr2* expression 1 day after injury, which is also associated with a significantly increased expression of *Tnfa* and *il1b*, which points towards a wave of pro-inflammatory macrophages being recruited to the heart shortly after injury (Supplementary Figure S9). Similarly, in adult mice after myocardial infarction, there is a significant influx of *Ccr2*-expressing macrophages at 3 days post injury, which coincides with an increase in *Il1b* expression (Supplementary Figure S9). Our own data indicate that during cardiac regeneration in adult zebrafish, *ccr2* was expressed (albeit non-significantly) at low levels 3 days after injury, predominantly in the recruited MC2 population (Supplementary Figure S9). Although it appears from our data that resident macrophages (MC1) in the uninjured control samples expressed higher levels of *tnfa* and *il1b* than the macrophages that appear after amputation (Supplementary Figure S9), we cannot rule out the possibility of a rapid influx of pro-inflammatory macrophages at an earlier timepoint, as observed in neonatal mice. In support of this notion, the expression profiles for *tnfa* and *il1b* at 3 days post injury followed a similar pattern in both neonatal mice and adult zebrafish. In particular, there was a reduction in expression of both genes 3 days post injury compared to uninjured controls (Supplementary Figure S9). Further analysis will be required at earlier timepoints to fully decipher the macrophage inflammatory response during cardiac regeneration in zebrafish.

Our data also reveal a potential zebrafish-specific mechanism for modulating the response of macrophages to CXC motif ligand (CXCL) chemokine signals. In mammals, G protein-coupled CXC chemokine receptor 3 (CXCR3) signalling is required for recruiting macrophages to the site of injury/infection (Viswanathan and Tobin, 2020). Previous reports indicate that zebrafish possess two orthologs of this gene, *cxcr3.2* and *cxcr3.3*. Although Cxcr3.2 appears to be a functionally active receptor, Cxcr3.3 lacks the ability to activate downstream signalling pathways and hence acts as a scavenger of Cxcr3 ligands, effectively dampening down the Cxcr3.2 response (Sommer et al., 2020). Although all the MCs express *cxcr3.2, cxcr3.3* is particularly enriched in resident macrophages (MC1), which would presumably reduce their ability to respond to Cxcr3 ligands (Supplementary Figure S10A, B and Table S1).

MMPs are also well-established players in wound healing and regeneration and have been linked to a variety of processes that are necessary to resolve damaged tissue (Caley et al., 2015). Transcriptomic analysis of zebrafish heart regeneration has consistently identified an upregulated expression of various MMPs during this process (Lien et al., 2006; Jopling et al., 2012; Gamba et al., 2017). Furthermore, MMPs have been shown to be involved in zebrafish fin regeneration (Bai et al., 2005), newt limb regeneration (Vinarsky et al., 2005), and salamander limb regeneration (Park and Kim, 1999). We initially assessed MMP expression in the entire heart during cardiac regeneration by reverse transcription followed by quantitative polymerase chain reaction (RT–qPCR) and observed a dynamic response during this process, in particular the expression of *mmp2* and *mmp14a/b* (zebrafish possess two *mmp14* orthologs) increasing substantially (Figure 4B). Analysis of our scRNA-seq data indicates that *mmp14a/b* were predominantly expressed by fibroblasts and macrophages. In particular, *mmp14a* was restricted to the fibroblast population and absent in macrophages, while *mmp14b* appeared to be expressed by both populations (Figure 4C; Supplementary Table S1). Of particular interest, we also observed that the expression of *mmp14b* was significantly higher in MC2 macrophages compared to the MC1 and MC3 populations (Figure 4D). To confirm that macrophages express MMP14 during regeneration, we performed IHC for IB4, a marker for macrophages (Lai et al., 2017), and MMP14 on regenerating zebrafish and P1 neonatal mouse hearts (7 dpa). In this manner, we were able to clearly detect MMP14^+^ macrophages present during regeneration in both species (Figure 4E–L). To gain further insight into MMP14 expression by macrophages, we re-analysed two previously published scRNA-seq datasets from non-regenerating adult mice and regenerating neonatal mice after myocardial infarction (Supplementary Figure S11A–C). Interestingly, *Mmp14*^+^ macrophages were absent at baseline and 3 days post myocardial infarction in both adult and neonatal mice (Supplementary Figure S11B and C). This is in contrast with zebrafish, in which *mmp14b*-expressing macrophages were present in uninjured hearts and peaked at 3 dpa (Supplementary Figure S11A). To determine whether MMP14 is required for cardiac regeneration, we employed a specific inhibitor of this protein, NSC405020, which blocks the collagenolytic activity of MMP14 but not its ability to activate other targets such as MMP2 (Remacle et al., 2012). NSC405020 targets the PEX domain of mammalian MMP14 and has also been shown to effectively inhibit chicken MMP14 (Andrieu et al., 2020). To assess whether NSC405020 could also target zebrafish MMP14, we compared the homology of the PEX domain with human, mouse, and chicken. Our data indicate that 2 out of 3 conserved (human, mouse, and chicken) residues in the PEX domain, which are proximal to NSC405020, are conserved in both zebrafish Mmp14a and Mmp14b (Supplementary Figure S12). Furthermore, the third conserved serine residue is present, albeit shifted towards the N-terminal by one amino acid (Supplementary Figure S12). Histological analysis of cardiac sections taken from 30 dpa adult zebrafish indicated that NSC405020 treatment significantly impaired cardiac regeneration, resulting in the formation of a large fibrin/collagen scar (*n* = 9) (Figure 5A–F). To gain further insight into how regeneration is disrupted by MMP14 inhibition, we labelled proliferating cardiomyocytes with EdU and Mef2c. In this manner, we were able to determine that NSC405020-mediated MMP14 inhibition significantly impaired cardiomyocyte proliferation (Figure 5G–I). To determine whether MMP14 inhibition directly disrupts the macrophage regenerative response, we repeated these experiments at 3 dpa, a timepoint when M2/MMP14^+^ macrophages appear. IHC analysis of cardiac sections revealed substantial number of macrophages proximal to the wound region at 3 dpa in untreated control samples (*n* = 5) (Figure 5J–P). Strikingly, the number of macrophages proximal to the wound region of NSC405020-treated samples was significantly reduced (*n* = 5) (Figure 5I–O), whereas the number of macrophages distal to the wound region was largely unaffected. This indicates that MMP14 inhibition impairs the ability of macrophages to migrate into the wound region during the process of regeneration. Based on these observations, we performed scRNA-seq analysis to assess what effect MMP14 inhibition had on the macrophage transcriptome. In order to identify the early events affected by NSC405020 treatment, we focused on 3 dpa when *mmp14b*-expressing macrophages appear. Following unbiased clustering of cells from either controls or NSC405020-treated samples, we were able to identify seven major cell populations (cardiomyocytes, endothelium, epicardium/EPDCs, lymphoid, myeloid, thrombocytes, and erythrocytes) (Figure 6A and B). We next compared cell type-specific gene expression profiles between the control and NSC405020-treated samples. Interestingly, the macrophage population (myeloid) displayed the highest number of genes whose expression was significantly affected by MMP14 inhibition (Figure 6C). Furthermore, analysis of the fibroblast population (the only other cell type to express *mmp14*) within this dataset indicates that, overall, there are no dramatic changes in the expression of genes associated with the fibrotic response following inhibitor treatment (Supplementary Figure S13A–D). Within the macrophage population, we could identify two clusters of macrophages, MC2a and MC2b, which were enriched for *mmp14b* and *cd9b* (Figure 6D; Supplementary Figure S14A). Furthermore, we were able to observe a robust upregulation of a number of genes (*grn1, fabp11a, lgals9l, ccl34b.1, g0s2*, and *lgals3bpb*), all of which have previously been associated with regenerating macrophages (Mitchell et al., 2019; Supplementary Figure S14B–G). Of potential interest, the heparin-binding epidermal growth factor-like growth factor (HB-EGF), encoded by *hbegfb*, which is cleaved and activated by MMP14 (Stawowczyk et al., 2017), is specifically upregulated in MC2 macrophages following inhibitor treatment (Figure 6E). Taken together, our data indicate that the macrophage response to cardiac injury in adult zebrafish is reminiscent of the early response observed in neonatal mice after myocardial infarction. Furthermore, we have determined that MMP14 is required for effective cardiac regeneration and that inhibiting the collagenolytic activity of MMP14 results in defective migration of M2 macrophages into the wound region.

**Figure 5:**
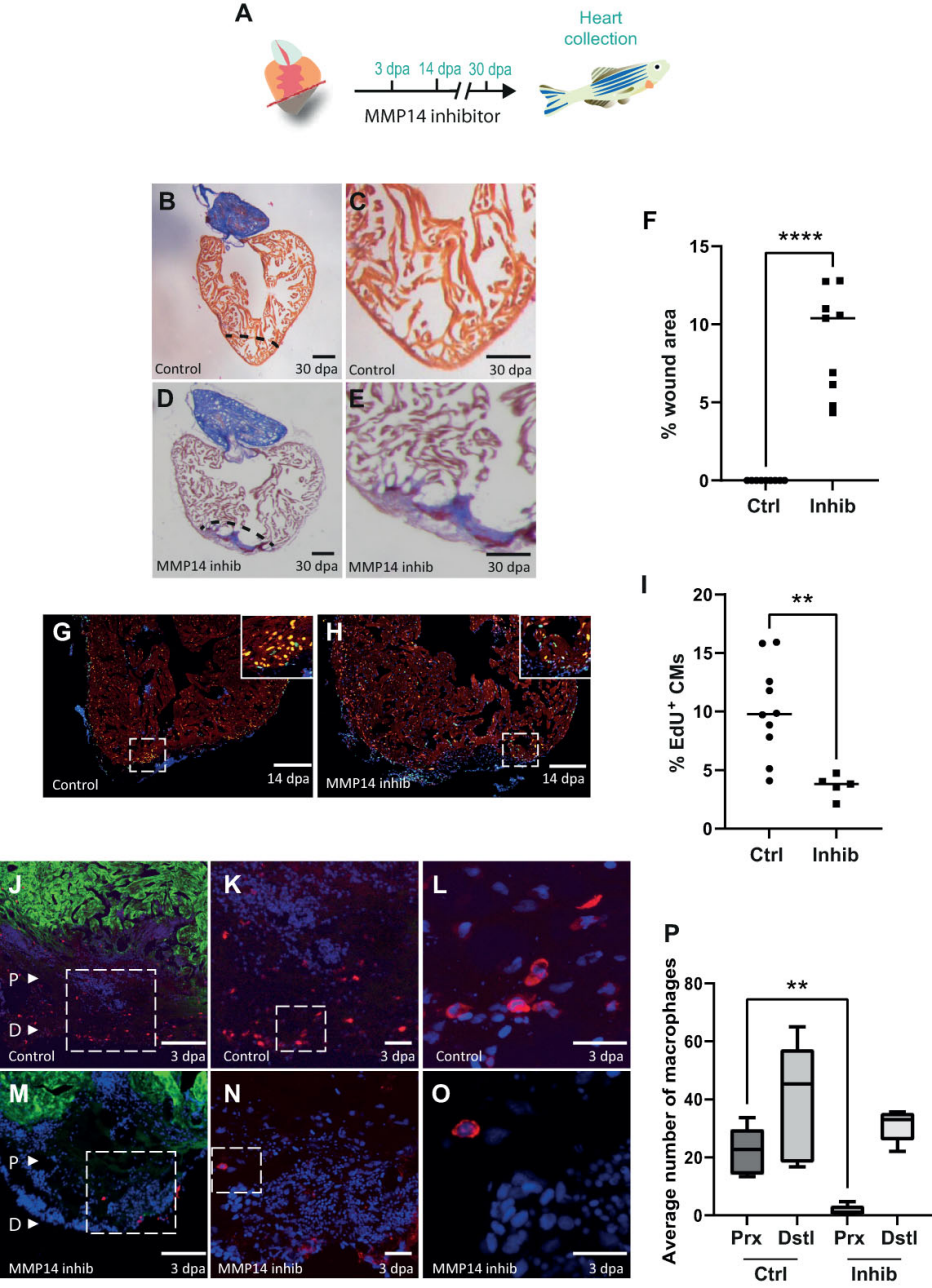
MMP14-expressing macrophages are required for cardiac regeneration. (**A**) Schematic of the inhibitor treatment protocol. (**B** and **D**) AFOG staining of a control (**B**) or NSC405020-treated (**D**) heart at 30 dpa. The black dashed line indicates the plane of amputation. Scale bar, 200 μm. (**C** and **E**) The same images as in **B** and **D** are shown at higher magnification, respectively. Note the presence of a large fibrin (red)/collagen (blue) scar. Scale bar, 100 μm. (**F**) The graph indicates the average area of the wound region as a percentage of the entire ventricle (*n* = 9). (**G** and **H**) EdU labelling of cycling cardiomyocytes in a control (**G**) or NSC405020-treated (**H**) heart. MEF2/cardiomyocytes (red), EdU (green). The dashed white box indicates the area shown at higher magnification in the inset. Scale bar, 200 μm.

**Figure 5:**
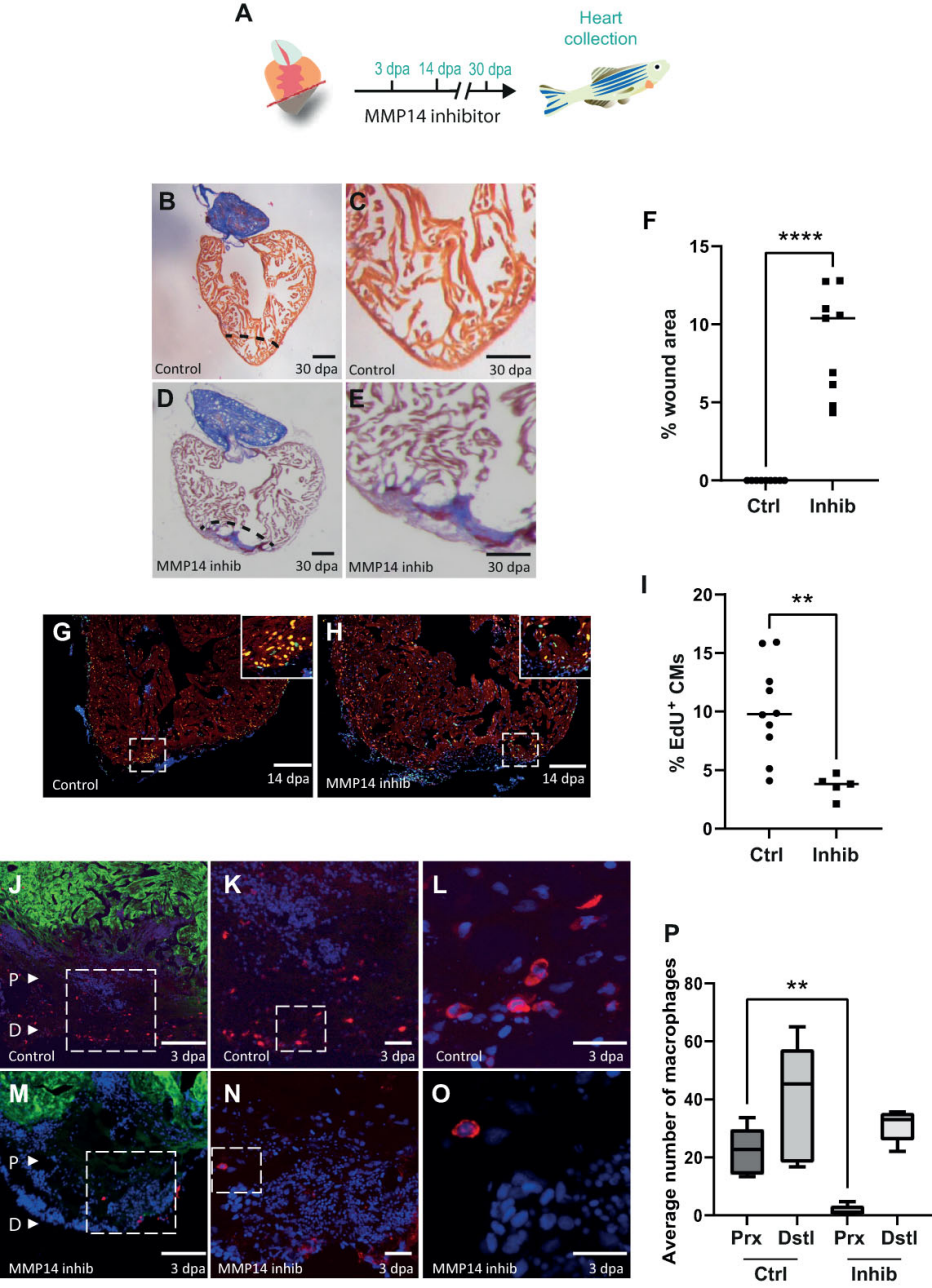
*(Continued)* (**I**) The graph indicates the percentage of EdU^+^ cardiomyocytes in the entire ventricle (control *n* = 10, inhibitor *n* = 5). (**J**–**O**) IHC on a 3 dpa control (**J**–**L**) or NSC405020-treated (**M**–**O**) zebrafish heart section for cardiomyocytes (GFP, green) and macrophages (IB4, red). (**J** and **M**) The letter P and the white arrow head point to proximal regions where macrophages were counted. The letter D and the white arrow head point to distal regions where macrophages were counted. Scale bar, 200 μm. (**K** and **N**) The regions highlighted by the dashed white box in **J** and **M** are magnified, respectively, which show macrophages in the wound region. Scale bar, 20 μm. (**L** and **O**) The regions highlighted by the dashed white box in **K** and **N** are magnified, respectively. Scale bar, 20 μm. (**P**) The graph indicates the average number of macrophages counted in either the proximal (Prx) or distal (Dstl) regions in 3 dpa control (Ctrl) or NSC405020-treated (Inhib) hearts. *P*-values were calculated using a non-parametric Mann–Whitney test. *P < 0.1, **P < 0.01, ***P < 0.001, ****P < 0.0001.

**Figure 6:**
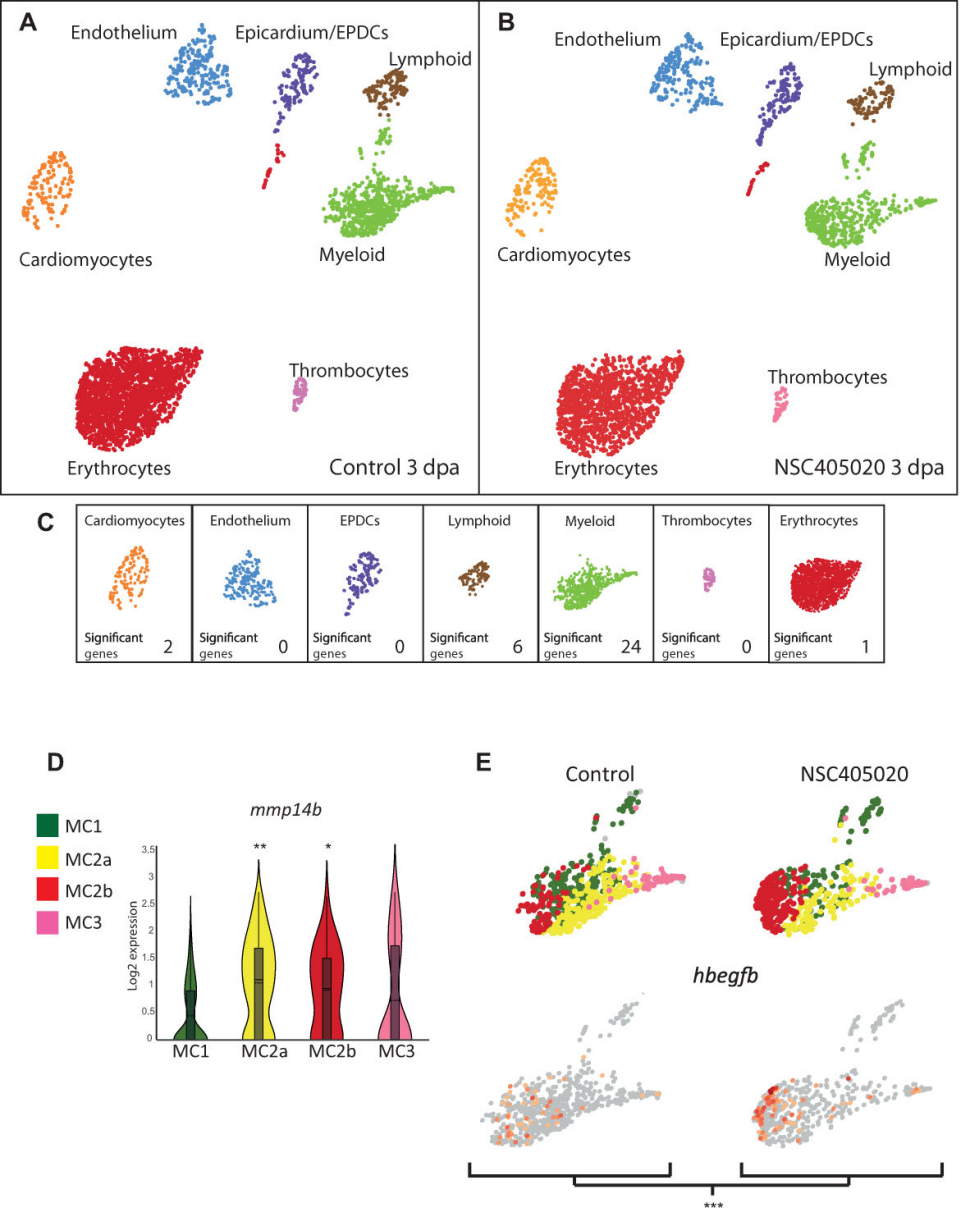
scRNA-seq analysis of control and MMP14 inhibitor-treated regenerating hearts. (**A** and **B**) UMAP clusters of the different populations of cells in control (**A**) and MMP14 inhibitor-treated (**B**) zebrafish hearts at 3 dpa. (**C**) The number of genes per cell type, which shows significantly differential expression between the control and MMP14 inhibitor-treated conditions (*P* < 0.1). (**D**) Violin plot compares the expression of *mmp14b* in MC1, MC2a, MC2b, and MC3 (*P < 0.1, **P < 0.05). (**E**) UMAP plots depict the relative expression of *hbegfb* in the different macrophage sub-clusters in control and MMP14 inhibitor-treated conditions (***P < 0.01).

## Discussion

Here, we report a detailed scRNA-seq-driven analysis of how interstitial cells behave during cardiac regeneration in zebrafish. Our data have uncovered a number of intriguing insights into the regenerative process and also highlighted notable differences between regenerating and non-regenerating species. Regarding the cellular composition of the adult zebrafish heart, when compared to the adult mouse heart, we found largely comparable proportions of endothelial cells and cardiomyocytes. However, we observed a notable difference in the proportion of cells derived from the epicardial lineage. In particular, the number of fibroblasts present in the adult zebrafish heart (1%–2%) was considerably lower than what has been reported for the adult mouse heart (11%) (Patra et al., 2017). In mammals, loss of cardiac tissue due to myocardial infarction triggers the differentiation of fibroblasts into myofibroblasts that are involved in replacing the lost tissue with a fibrotic scar (Humeres and Frangogiannis, 2019). Although this initial phase is essential for maintaining cardiac integrity, the fibrotic response can spread throughout the heart leading to impaired cardiac function and ultimately failure. Indeed, it has been proposed for some time that the difference between scarring and regeneration could be influenced by the fibrotic response to injury (Hara et al., 2017). One feature of this response involves *Acta2*, a gene commonly restricted to smooth muscle cells for encoding α-smooth muscle actin, which becomes specifically upregulated in myofibroblasts (Humeres and Frangogiannis, 2019). Our data, combined with re-analysis of scRNA-seq data from mouse models, showed that, following injury, the epicardial/fibroblast lineage strongly upregulated a number of myofibroblast-associated genes such as *Postn/postnb* and *Fn1*/*fn1a* in both mouse and zebrafish. However, this was not the case for *Acta2*, which was upregulated in non-regenerating P8 neonates and adult mouse, relatively weakly upregulated in regenerating P1 neonatal mouse and low/absent in zebrafish. This could indicate a reduction in myofibroblast differentiation, or the nature of this transition, and potentially the associated fibrotic response. Another possibility involves the ability of Acta2 to enable myofibroblasts to contract during the process of wound healing (Ibrahim et al., 2015). Indeed, wound contraction following injury serves to decrease the amount of tissue that needs to be repaired. In the heart, this would allow the damaged tissue to be repaired rapidly in order to avoid rupture, a process that most likely supersedes regeneration in adult mammals. It is therefore conceivable that in regenerating tissue, wound contraction may not be required and could even inhibit regeneration by impeding certain regenerative processes, such as neovascularization, which may benefit from an open/relaxed wound environment. Further research will be required in order to determine whether the lack of Acta2 observed in zebrafish fibroblasts can be linked to reduced myofibroblast differentiation and fibrosis, or whether this affects a specific feature of myofibroblasts, such as contractility. We also acknowledge that a possible limitation is the cardiac resection injury model used in this study, which may not induce a comparable fibrotic response as that observed in adult mammals after myocardial infarction.

Our data also highlight that the choice of genetic marker used to isolate and study fibroblasts is critical. Indeed, we found that genes that have previously been reported to be upregulated in fibroblasts in zebrafish, such as *rspo1* (Sanchez-Iranzo et al., 2018), were in fact expressed by *tcf21^–^* neural crest derivatives. The origin of *rspo1^+^;tcf21^–^* cells in the epicardial layer and whether they play a role in cardiac regeneration will require further investigation.

Following cardiac injury in adult zebrafish, there is a rapid endothelial response resulting in wound neovascularization. This precedes the expansion of proliferating cardiomyocytes, which will repopulate and ultimately regenerate the missing tissue. Our data indicate that *tal1* plays an essential role in the endothelial regenerative response and that inhibition of Tal1 by expressing a DN isoform significantly disrupts cardiac regeneration leading to the formation of an extensive fibrin/collagen scar. We have also shown that inhibiting Tal1 reduces the number of proliferating cardiomyocytes. Whether this is a direct or indirect effect will require further investigation. Lastly, our data also indicate that expressing DN Tal1 results in a failure of wound re-vascularization. This is presumably the primary effect caused by inhibiting Tal1 during regeneration, which subsequently impacts cardiomyocytes, which are unable to proliferate and repopulate a wound that has not been re-vascularized. Tal1 has been linked to a number of endothelial processes that may affect the regenerative response, such as the regulation of endocardial cell–cell contacts, endocardial identity, and neovascularization (Tang et al., 2006; Schumacher et al., 2013). Although *tal1* expression does not increase dramatically during cardiac regeneration, based on previous research, it is reasonable to speculate that the genetic programs that Tal1 regulates are, very likely, dictated by its co-factors (Stanulovic et al., 2017). Indeed, a combination of chromatin immunoprecipitation and transcriptomic analysis has shown that, in the absence of *Lmo2*, TAL1 relocates to different DNA target sites where it regulates alternative genetic programs. Our scRNA-seq data indicate that the expression of the Tal1 co-factor, *lmo2*, decreases in *tal1^+^* endothelial cells at 7 dpa. Intriguingly, we also observed an increase in the expression of the tight junction-associated gene *cgnl1* at 7 dpa in *tal1^+^* endothelial cells. *Cgnl1* has also been implicated in neovascularization (Chrifi et al., 2017). Because Tal1 has previously been described to regulate endothelial tight junctions during endocardial development, it is tempting to speculate that this process has been disrupted following Tal1 inhibition, resulting in defective re-vascularization and impaired cardiac regeneration.

Macrophages are key regulators of regeneration, and evidence for their involvement in cardiac regeneration has been demonstrated in a variety of different species (Aurora et al., 2014; Godwin et al., 2017; Lai et al., 2017). Our scRNA-seq data have revealed a number of interesting features associated with macrophages during cardiac regeneration. Firstly, although resident macrophages in the uninjured heart appear to have a more pro-inflammatory signature compared to the population that appears at 3 dpa, which is also in agreement with another recent study (Ma et al., 2021), we cannot rule out the possibility that the situation may be reversed at an earlier timepoint, as the case in regenerating neonatal mouse hearts. Previous reports have indicated that during regeneration in neonatal mice, there is no recruitment of pro-inflammatory *Ccr2*-expressing macrophages (Lavine et al., 2014). However, analysis of published scRNA-seq data indicates a rapid influx of *Ccr2*-expressing macrophages 1 day post myocardial infarction coincident with elevated *Tnfa* and *Il1b* expression, an event that may have been missed previously. Furthermore, at 3 days post myocardial infarction in regenerating neonatal mouse hearts, despite being more numerous, macrophages expressed lower levels of *Tnfa* and *Il1b* compared to sham-operated animals. We observed a similar increase in macrophage cell number but reduction in *tnfa* and *il1b* expression within these cells in zebrafish hearts 3 days after injury. These data suggest that the inflammatory response of macrophages in regenerating adult zebrafish hearts is more reminiscent of the regenerating neonatal mouse macrophage response; however, future studies at earlier timepoints will be required to confirm these observations. Interestingly, in regenerating zebrafish hearts, we could also detect a population of proliferating macrophages. Similarly, proliferating macrophages have been observed in mice post myocardial infarction, which serves to maintain macrophage numbers during the injury response (Sager et al., 2016). More focused studies will be required in order to determine whether the initial macrophage response, occurring during the first hours/days after injury, reflects any difference in regenerative capacity between adult zebrafish and mammals.

Our data also indicate that zebrafish possess a potentially unique mechanism for regulating the macrophage response to CXCL chemokine signals. CXCR3 and its ligands are responsible for recruiting macrophages to sites of injury/infection (Torraca et al., 2015). Previous research indicates that zebrafish possess two CXCR3 orthologs, Cxcr3.2 that is a functional G protein-coupled receptor and Cxcr3.3 that lacks downstream signalling capabilities. It is apparent that Cxcr3.3 acts as ligand scavenger, reducing the amount of Cxcr3 ligands available to bind to and activate Cxcr3.2 (Sommer et al., 2020). Our data indicate that during cardiac regeneration in zebrafish, all macrophages express *cxcr3.2* in relatively equal proportions; however, resident macrophages also express nearly 2-fold more *cxcr3.3* than recruited macrophages and, as such, it is fair to assume that this will reduce their ability to respond to Cxcr3 ligands. This may provide zebrafish with an elegant mechanism for fine-tuning the resident M1 macrophage response to Cxcr ligands. For example, modulating Cxcr3 signalling may play a role in blunting the inflammatory response of the M1 resident macrophages during cardiac regeneration. It could also potentially serve to reduce the mobility of resident macrophages and help to maintain this population within the heart.

MMPs have frequently been associated with cardiac injury and regeneration. In particular, MMP14 appears to be the major type 1 collagenase in ischemic mouse hearts (Koenig et al., 2012). Our data indicate that Mmp14b is particularly enriched in macrophages that appear at 3 dpa. Furthermore, we observed that inhibiting the collagenolytic activity of MMP14 resulted in defective migration of macrophages into the injury site and a subsequent failure to regenerate the myocardium, leading to the formation of large collagen/fibrin scar. Although the fibroblast population also expresses *mmp14a/b* and could potentially be affected by NSC405020 treatment, a previous study indicates that deleting fibroblasts does not significantly impact the overall regenerative process in adult zebrafish (Sanchez-Iranzo et al., 2018). In agreement with this, our scRNA-seq analysis of MMP14-inhibited regenerating hearts indicates that the macrophage population displays the highest number of differentially expressed genes, while the fibro-blast population is unaffected. Taken together, this suggests that the effects we observe on cardiac regeneration following NSC405020 treatment are largely due to inhibiting Mmp14b in the macrophage population. One potentially interesting gene deregulated specifically in macrophages following MMP14 inhibition is *hbegfb*, which has been linked to a variety of processes important for cardiac regeneration (Tanaka et al., 2002; Iwamoto and Mekada, 2006). However, expression of *hbegfb* appears to increase when cardiac regeneration fails following MMP14 inhibition. In this context, HB-EGF has previously been shown to have detrimental effects on the remodeling process after myocardial infarction in mammals by enhancing fibroblast activation and invasion (Ushikoshi et al., 2005). It could therefore play a role in the development of the substantial fibrin/collagen scar that is present at 30 dpa when MMP14 is inhibited. Re-analysis of scRNA-seq data from adult mice and neonates following myocardial infarction showed that, conversely to what we observed in zebrafish, *Mmp14*-expressing macrophages were not present in the myocardium prior to injury. Whether the presence of Mmp14b-producing resident macrophages confers zebrafish with some kind of regenerative advantage will require further investigation. However, in stark contrast to our own observations in zebrafish, it appears that in adult mice, MMP14 plays a deleterious role after cardiac injury (Koenig et al., 2012). Indeed, MMP14 heterozygote knockout mice display a marked improvement in survival post myocardial infarction due to reductions in infarct size, left ventricular dilation, and compensatory hypertrophy. Furthermore, there is also a significant reduction in the number of macrophages localized to the infarct area in MMP14^+/–^ mice, similar to our own observations in regenerating zebrafish hearts when Mmp14 is inhibited. More recently, it has been shown that macrophage-specific deletion of MMP14 in adult mice reduces left ventricular dysfunction following myocardial infarction (Alonso-Herranz et al., 2020). It appears that loss of MMP14 in macrophages results in attenuated, TGFβ-dependent fibrosis. Our own data indicated that *mmp14b*-expressing macrophages are recruited to the wound site a few days after injury, similar to observations in mouse myocardial infarction models. Targeting Mmp14 in zebrafish with a specific inhibitor disrupted cardiac regeneration, which appears to be the complete inverse of the situation described in adult mice. Why this is the case remains unclear. It is possible that zebrafish Mmp14b possesses properties that are absent from mammalian MMP14, or this could be due to the expression of *mmp14b* by resident macrophages in uninjured zebrafish hearts, a feature that is not present in neonatal or adult mouse hearts. It may also be the case that MMP14 macrophages are performing a similar role in adult mouse hearts following injury but another pro-regenerative process is absent. A failure to co-ordinate a multi-faceted regenerative response could in fact be detrimental. Future research will be required to determine why MMP14 plays a positive pro-regenerative role in adult zebrafish and yet appears to exacerbate the damage associated with myocardial infarction in mammals.

In summary, our data have highlighted a number of intriguing features of the interstitial cellular response during cardiac regeneration in adult zebrafish. Although there are notable differences when compared to non-regenerating mammalian hearts, there are also many similarities, which certainly offers hope that we will eventually be able to recapitulate this process in adult humans.

## Materials and methods

### Zebrafish transgenic lines and husbandry

Zebrafish were maintained under standardized conditions and experiments were conducted in accordance with local approval (APAFIS#4054-2016021116464098 v5) and the European Communities council directive 2010/63/EU. Embryos were staged as described (Kimmel et al., 1995). *Tg(fli1a:GFP)y1* was provided by the Centro de Medicina Regenerativa de Barcelona (CMRB). *Tg(gata1:DsRed2)sd2* and *Tg(mpeg1.1:mCherryF)ump2* were provided by the Lutfalla lab, University of Montpellier. *Tg(col1a2:loxP-mCherry-NTR)cn11* was provided by the Mercader lab, Bern University. *Tg(eab2:[EGFP-T-mCherry])vu295* was provided by the Chen lab, Vanderbilt University Medical Center. *Tg(tcf21:DsRed2)pd37* was provided by the Poss lab, Duke University. All larvae and adults were euthanised by administration of excess anaesthetic (Tricaine).

### Zebrafish cardiac regeneration

All amputations were performed as described (Jopling et al., 2010), in accordance with local approval (APAFIS#4052). For the scRNA-seq analysis, we used 6-month-old sibling offspring from an incross of *Tg(cmlc2a:GFP)*, which were generated on an AB wild-type background.

## Supplementary Material

mjac059_Supplemental_Files
