# Supplementary material for: The regenerative response of cardiac interstitial cells
Source: J Mol Cell Biol. 2022 Oct 22;14(10):mjac059. doi: 10.1093/jmcb/mjac059 (PMC10068904; doi:10.1093/jmcb/mjac059)
Supplement: mjac059_Supplemental_Files [file mjac059_supplemental_files.zip › Supplemental files.pdf]

Supplemental figures

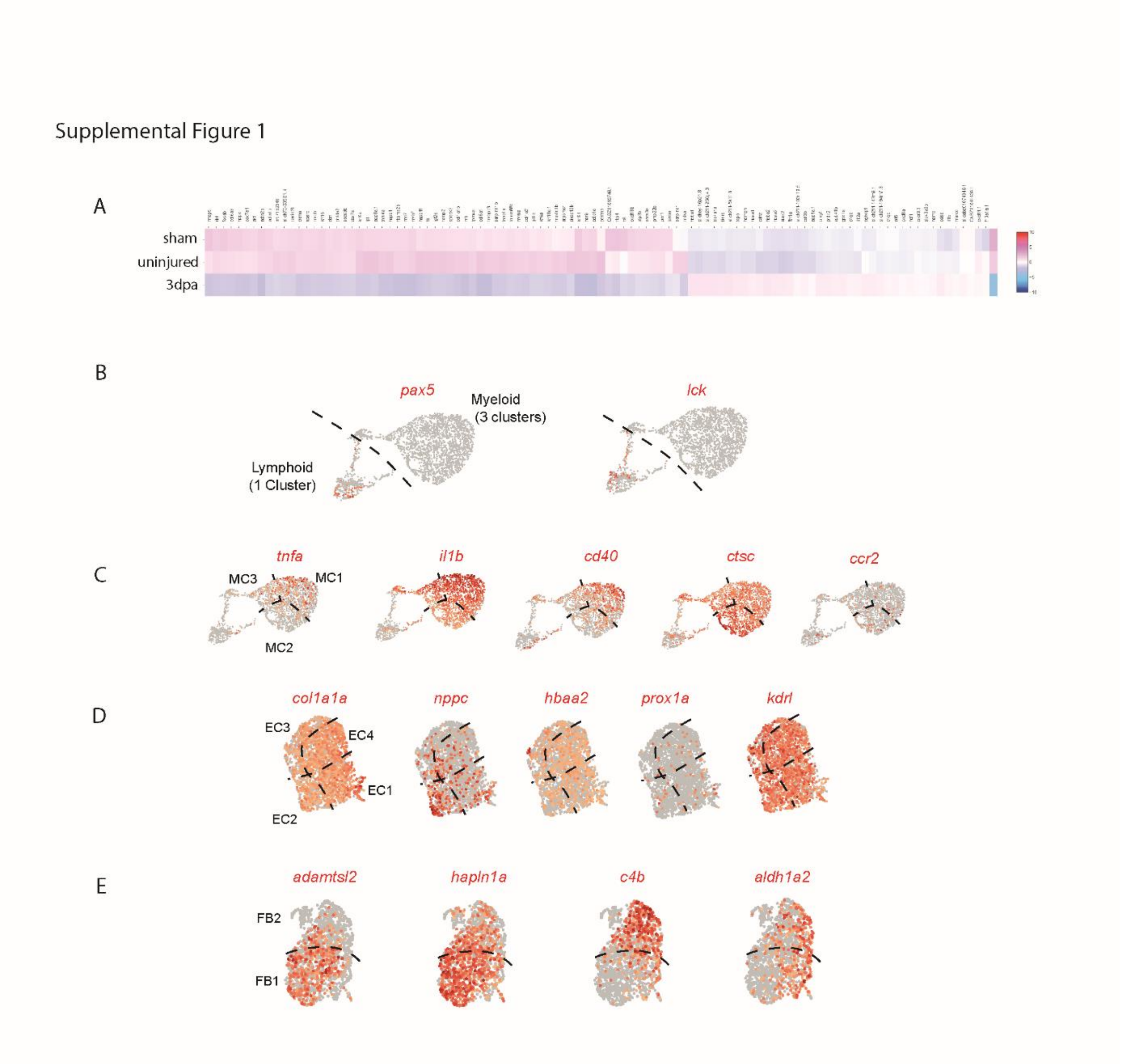

Supplemental figure S1. Single cell RNAseq analysis of regenerating zebrafish hearts. (**A**) Heat map depicting differentially expressed genes among sham operated, uninjured and 3dpa samples. (**B-E**) UMAP plots depicting the relative expression of particular genes within the different sub clusters. (**B**) Lymphoid cells (*pax5* and *lck*). (**C**) Macrophages (*tnfa*, *il1b*, *cd40*, *ctsc*, *ccr2*). (**D**) Endothelium (*col1a1a*, *nppc*, *hbaa2*, *prox1a*, *kdrl*). (**E**) Fibroblasts (*adamtsl1*, *hapln1a*, *c4b*, *aldh1a2*).

Supplemental Figure 2

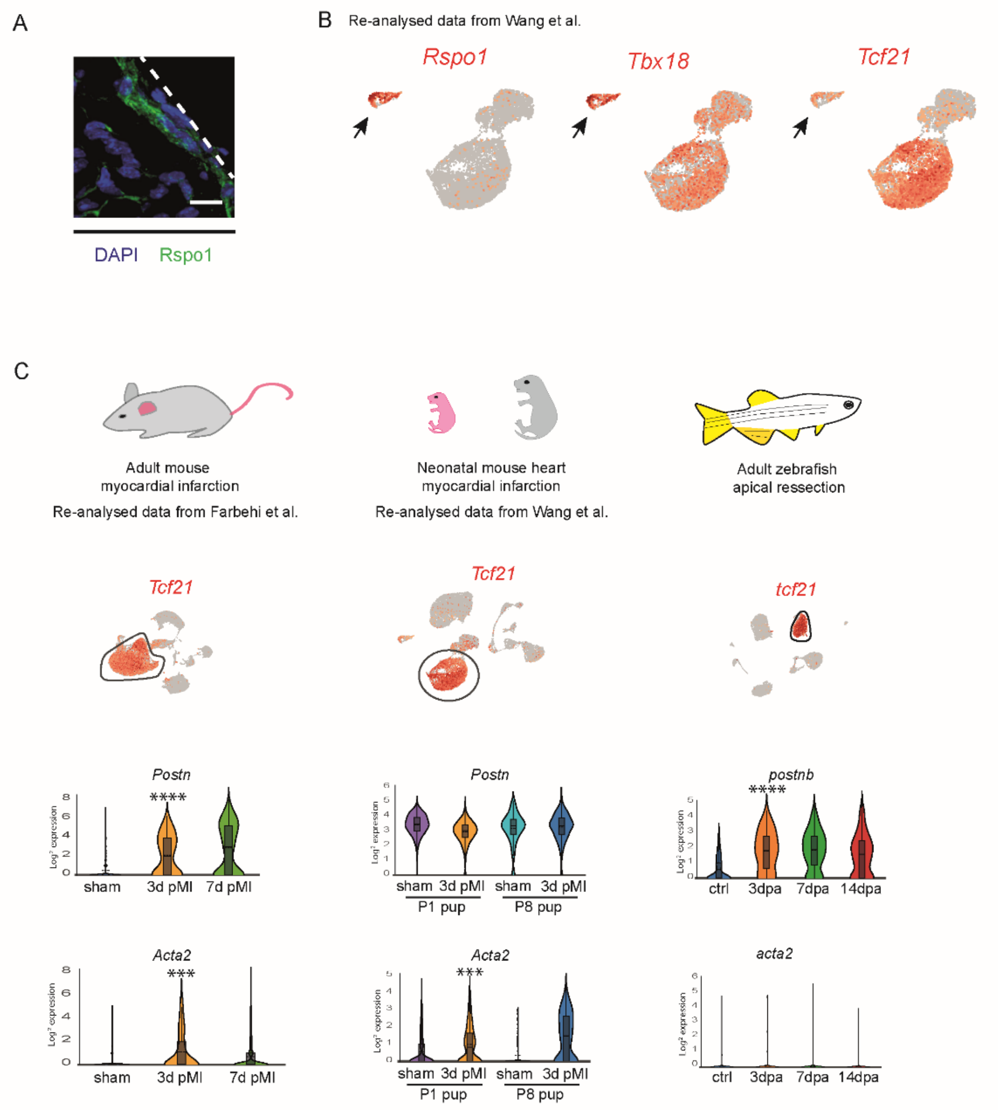

Supplemental figure S2. Mesenchymal lineages and cardiac fibroblast activation in the regenerating zebrafish heart. (**A**) IHC image of 7dpa neonatal mouse heart labelled with RSPO1 antibody (green), white dashed line indicates the outer edge of the epicardium (scale bar 10µm). (**B**) UMAP plots from re-analysed neonatal mouse scRNA-seq data (*Wang et al.)* depicting the co-expression of *Rspo1* with *Tbx18* and *Tcf21*. Note that although *Rspo1* is co-expressed with both genes in a smaller cluster (black arrow) there is relatively little expression within the main *Tcf21*$^{+}$ fibroblast cluster. (**C**) Re-analysis of scRNA-seq data from adult (Farbehi *et al.)* and neonatal mice (Wang et al.)

after myocardial infarction compared with adult zebrafish after apical resection. Adult mouse data is from 3 and 7 days post myocardial infarction (MI). Neonatal is from postnatal day 1 and postnatal day 8 pups, with sham and 3 days post MI samples. UMAP plots highlight the fibroblast population (*Tcf21*) in each dataset. (**D**) Violin plots represent the expression of *Periostin* and *Acta2* at different time points after injury in each dataset. Note- *Periostin* is expressed in uninjured neonatal mouse hearts, *Acta2* is expressed by fibroblasts in both adult and neonatal mice after injury but not by adult zebrafish fibroblasts. (***$P<0.01$, **** $P<0.001$).

Supplemental Figure 3

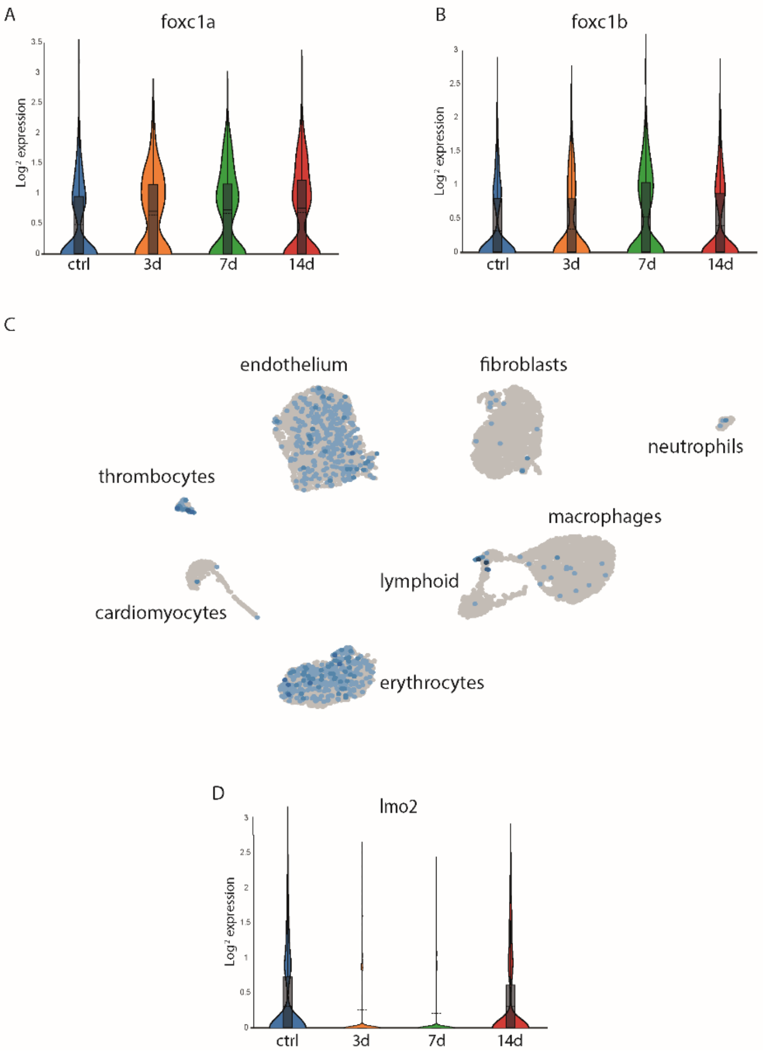

Supplemental figure S3. Analysis of endothelial developmental transcription factors during cardiac regeneration. (**A**, **B**) Violin plots comparing the expression of *foxc1a* (**A**) and *foxc1b* (**B**) at different time points after injury (uninjured (ctrl), 3dpa, 7dpa and 14dpa) within the endothelial population of cells. (**C**) UMAP plot of the different cell clusters in which *tal1* is expressed (blue dots). (**D**) Violin plot comparing the expression of *lmo2* at different time points after injury (uninjured (ctrl), 3dpa, 7dpa and 14dpa) within the endothelial population of cells.

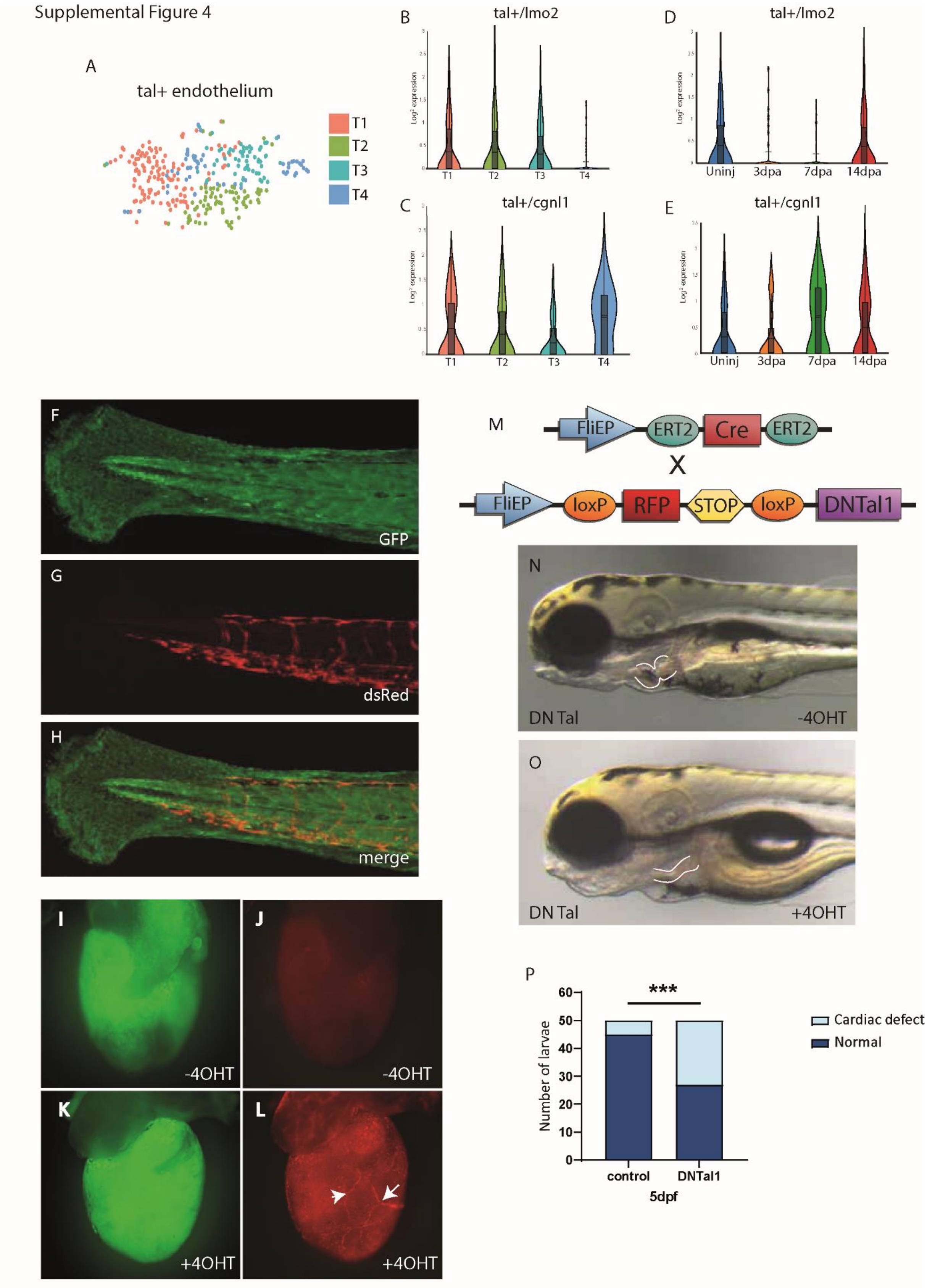

Supplemental figure S4. Tal1 is a regulator of the endothelial regenerative response. (**A**) UMAP plot of re-clustered *tal1* expressing cells. Coloured boxes represent the different clusters within this

population (T1-T4). (**B-E**) Violin plots indicating the expression of either *lmo2* or *cgnl1* in each cluster (**B**,**C**) and at each time point during regeneration (**D**,**E**). (**F-L**) Fluorescent images of a *Tg(eab2:[EGFP-T-mCherry])*^{vu295}/*Tg(fliEP:Ert2CreErt2)* 5dpf larvae and adult hearts treated with tamoxifen. (**F**) GFP is ubiquitously expressed. (**G**) mCherry is expressed in endothelial cells following tamoxifen induced Cre mediated recombination. (**H**) Merged image of F and G. Note the vasculature is labelled with mCherry (**G**) indicating that Ert2CreErt2 in *Tg(fliEP:Ert2CreErt2)* zebrafish is restricted to endothelial cells. (**I**) GFP is ubiquitously expressed in the adult heart. (**J**) In the absence of tamoxifen (-4OHT) mCherry is not expressed in endothelial cells. (**K**) GFP is ubiquitously expressed in the adult heart. (**L**) In the presence of tamoxifen (+4OHT) mCherry is expressed in endothelial cells, white arrow heads point to coronary vasculature. (**M**) Diagram indicating the 2 constructs used to generate the *Tg*(*fliEP:Ert2CreErt2;fliEP:loxRFPlox:DNtal*) transgenic zebrafish line. (**N,O**) *Tg*(*fliEP:Ert2CreErt2;fliEP:loxRFPlox:DNtal*) transgenic zebrafish larvae either untreated -4OHT (**N**) or tamoxifen treated +4OHT (**O**). The developing heart has been highlighted with white lines, note that the heart in the tamoxifen treated larvae has failed to develop normally. (**P**) Graph depicting the number of larvae which develop a cardiac defect in either untreated (control) or tamoxifen treated (DN Tal1) groups. Fisher's exact test, n=50 larvae for each condition (***: $p<0.001$).

Supplemental Figure 5

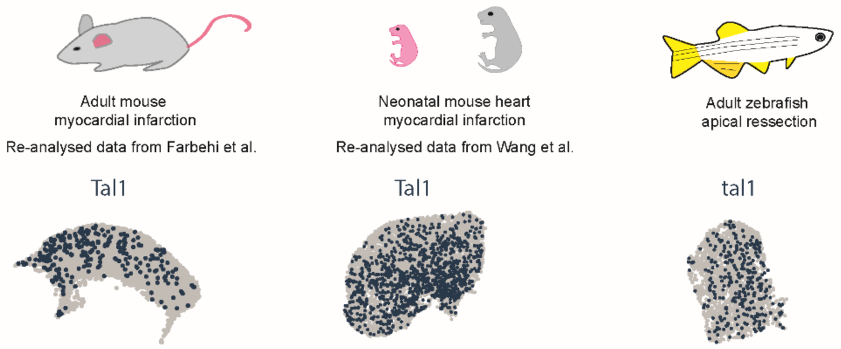

Supplemental figure S5. Tal1 is a regulator of the endothelial regenerative response. Re-analysis of scRNA-seq data from post myocardial infarction adult mice (Farbehi *et al*.) and neonatal mice (Wang *et al*.) compared with adult zebrafish after apical resection. UMAP plots indicating the expression of *Tal1* (dark blue dots) within the endothelial population of cells in each dataset.

Supplemental Figure 6

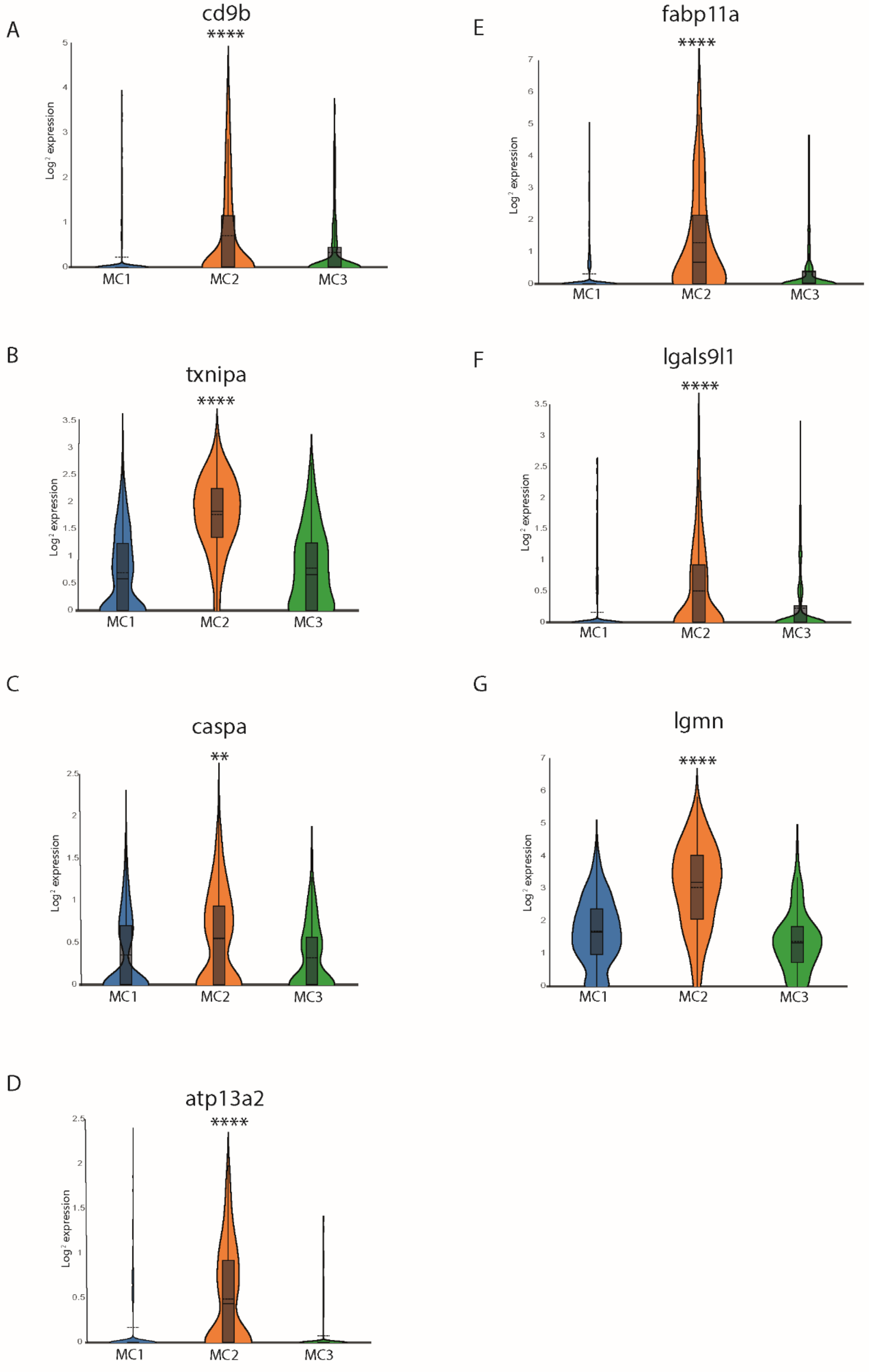

Supplemental figure S6. Genes enriched in the MC2 macrophage population. (**A-G**) Violin plots comparing the expression of different genes within the macrophage subpopulations (MC1, MC2 and MC3), *cd9b* (**A**), *txnipa* (**B**), *caspa* (**C**), *atp13a2* (**D**), *fabp11a* (**E**), *lgals9l1* (**F**) and *lgmn* (**G**). *P* values were adjusted using the Benjamini-Hochberg correction. *$P<0.1$, **$P<0.05$, ***$P<0.01$, **** $P<0.001$.

Supplemental Figure 7

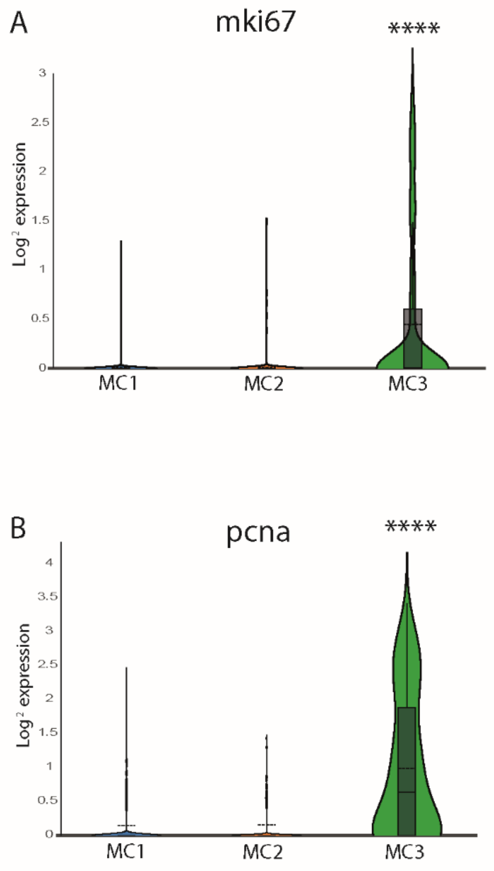

Supplemental figure S7. Genes enriched in the proliferating macrophage population. (**A,B**) Violin plots comparing the expression of proliferation associated genes within the macrophage subpopulations (MC1, MC2 and MC3), *mki67* (**A**) and *pcna* (**B**). *P* values were adjusted using the Benjamini-Hochberg correction. **P<0.1*, ***P<0.05*, ****P<0.01*, **** *P<0.001*.

Supplemental Figure 8

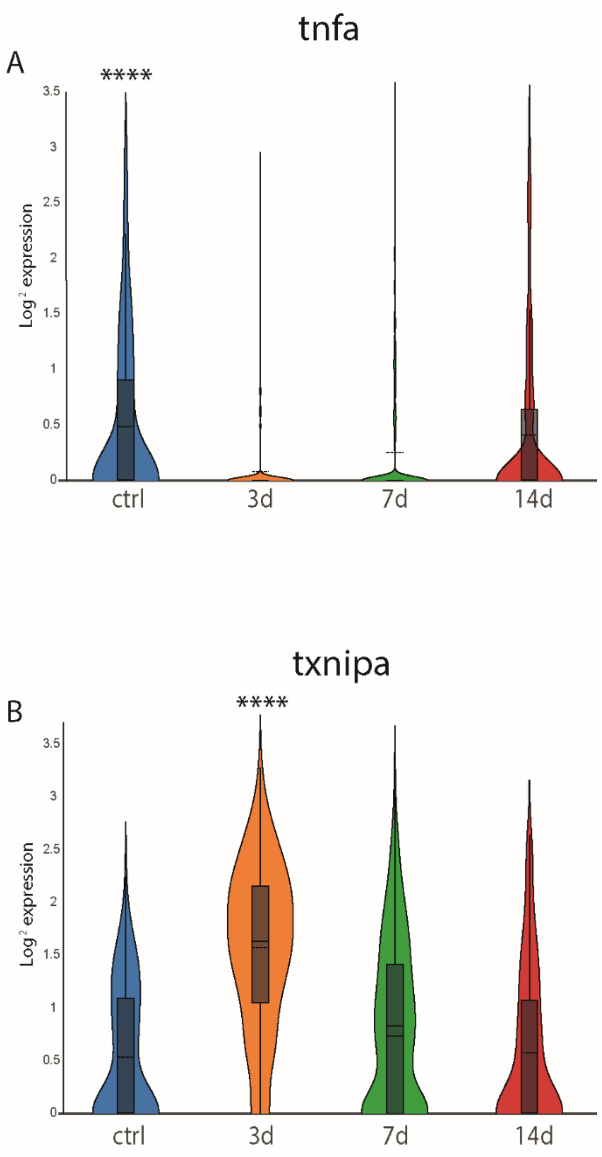

Supplemental figure S8. Dynamic expression of inflammatory genes in macrophages at different time points during cardiac regeneration. (**A**,**B**) Violin plots comparing the expression of inflammatory genes by macrophages at different time points during cardiac regeneration (uninjured (ctrl), 3dpa, 7dpa and 14dpa), *tnfa* (**A**), and *txnipa* (**B**). *P* values were adjusted using the Benjamini-Hochberg correction. *P<0.1, **P<0.05, ***P<0.01, **** P<0.001.

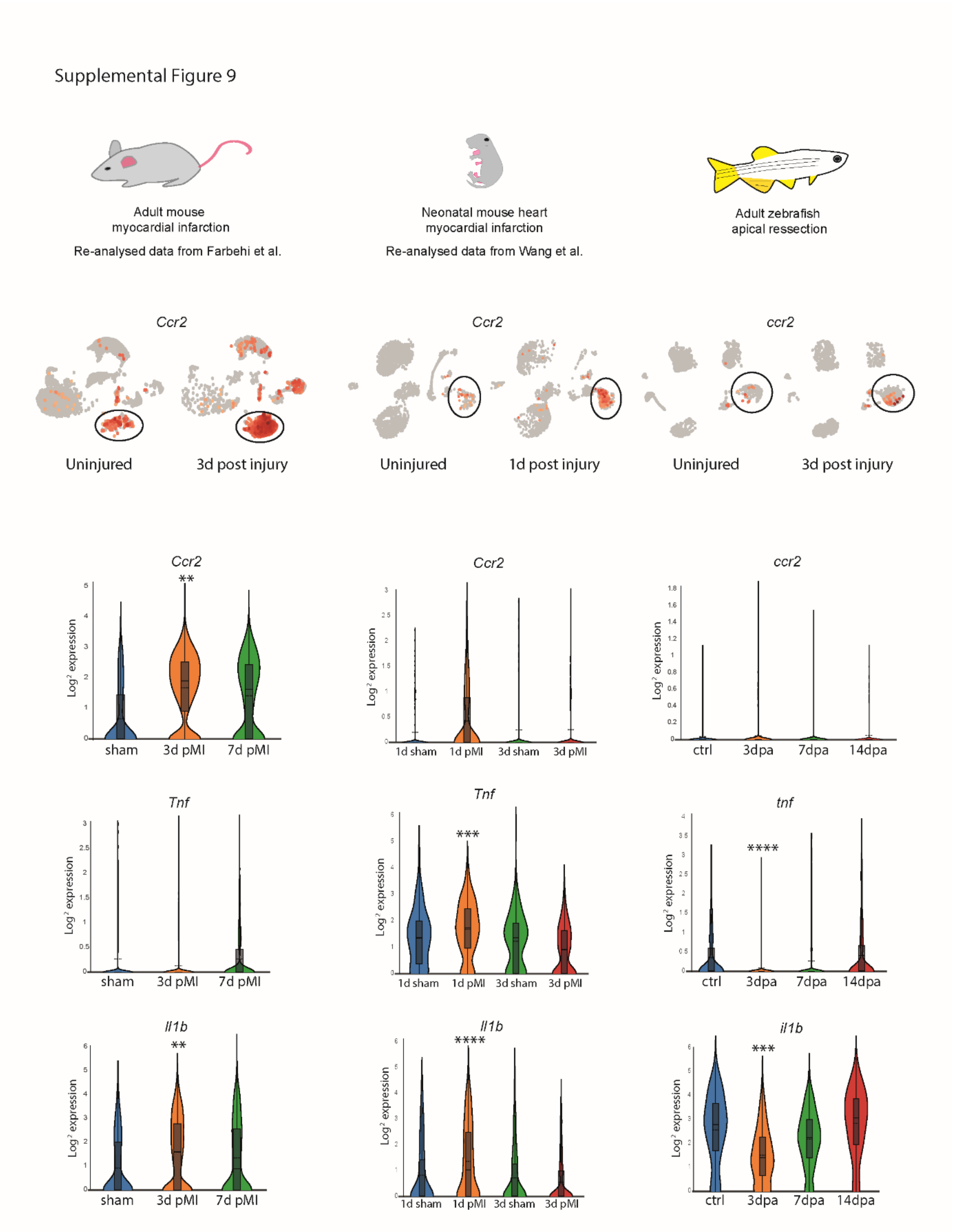

Supplemental figure S9. Comparison of the macrophage inflammatory response between species. *Adult mouse myocardial infarction.* Re-analysed UMAP plots depicting the expression of *Ccr2* in uninjured sham conditions vs 3 days post injury. Black circles indicate the macrophage population. Violin plots showing the expression of *Ccr2*, *Tnfa* and *Il1b* at different time points after injury (sham, 3

days post myocardial infarction (d pMI) and 7d pMI) in macrophages. *Neonatal mouse myocardial infarction.* Re-analysed UMAP plots depicting the expression of *Ccr2* in uninjured sham conditions vs 1 day post injury. Black circles indicate the macrophage population. Violin plots showing the expression of *Ccr2*, *Tnfa* and *Il1b* at different time points after injury (1 day sham, 1 day post myocardial infarction (d pMI) and 3d sham, 3d pMI) in macrophages. *Adult zebrafish apical resection*. UMAP plots depicting the expression of *Ccr2* in uninjured control conditions vs 3 days post injury. Black circles indicate the macrophage population. Violin plots showing the expression of *Ccr2*, *Tnfa* and *Il1b* at different time points after injury (control, 3 days post amputation (dpa) and 7dpa and 14dpa) in macrophages. *P* values were adjusted using the Benjamini-Hochberg correction. **P<0.1*, ***P<0.05*, ****P<0.01*, **** *P<0.001*.

Supplemental Figure 10

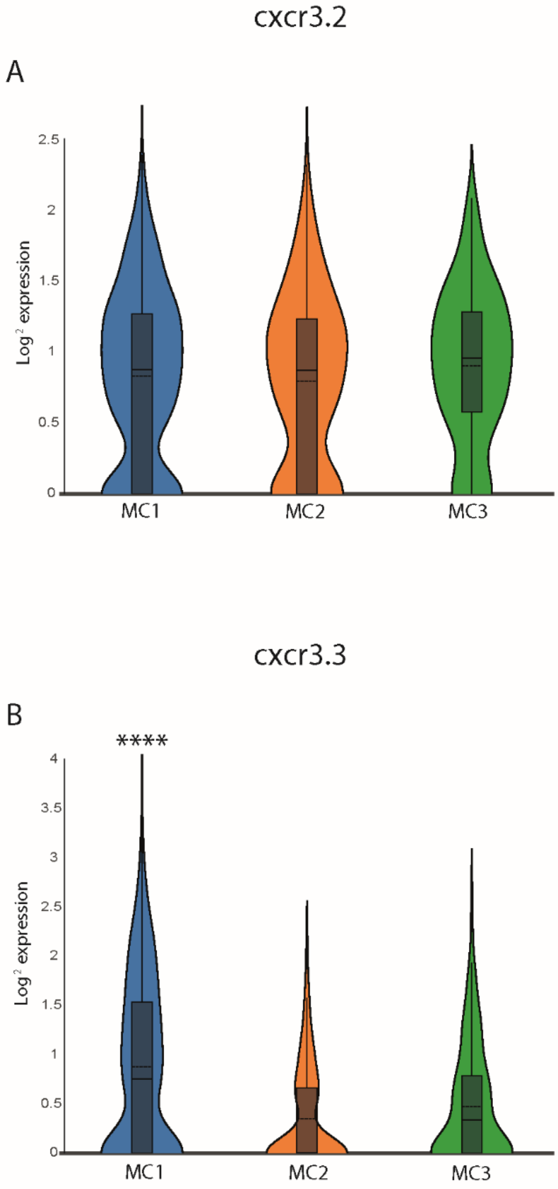

Supplemental figure S10. The expression of *cxcr3.2* and *cxcr3.3* within the macrophage subpopulations. (**A,B**) Violin plots comparing the expression of *cxcr3.2* and *cxcr3.3* within the macrophage subpopulations (MC1, MC2 and MC3), *cxcr3.2* (**A**) and *cxcr3.3* (**B**). *P* values were adjusted using the Benjamini-Hochberg correction. *$P<0.1$, **$P<0.05$, ***$P<0.01$, **** $P<0.001$.

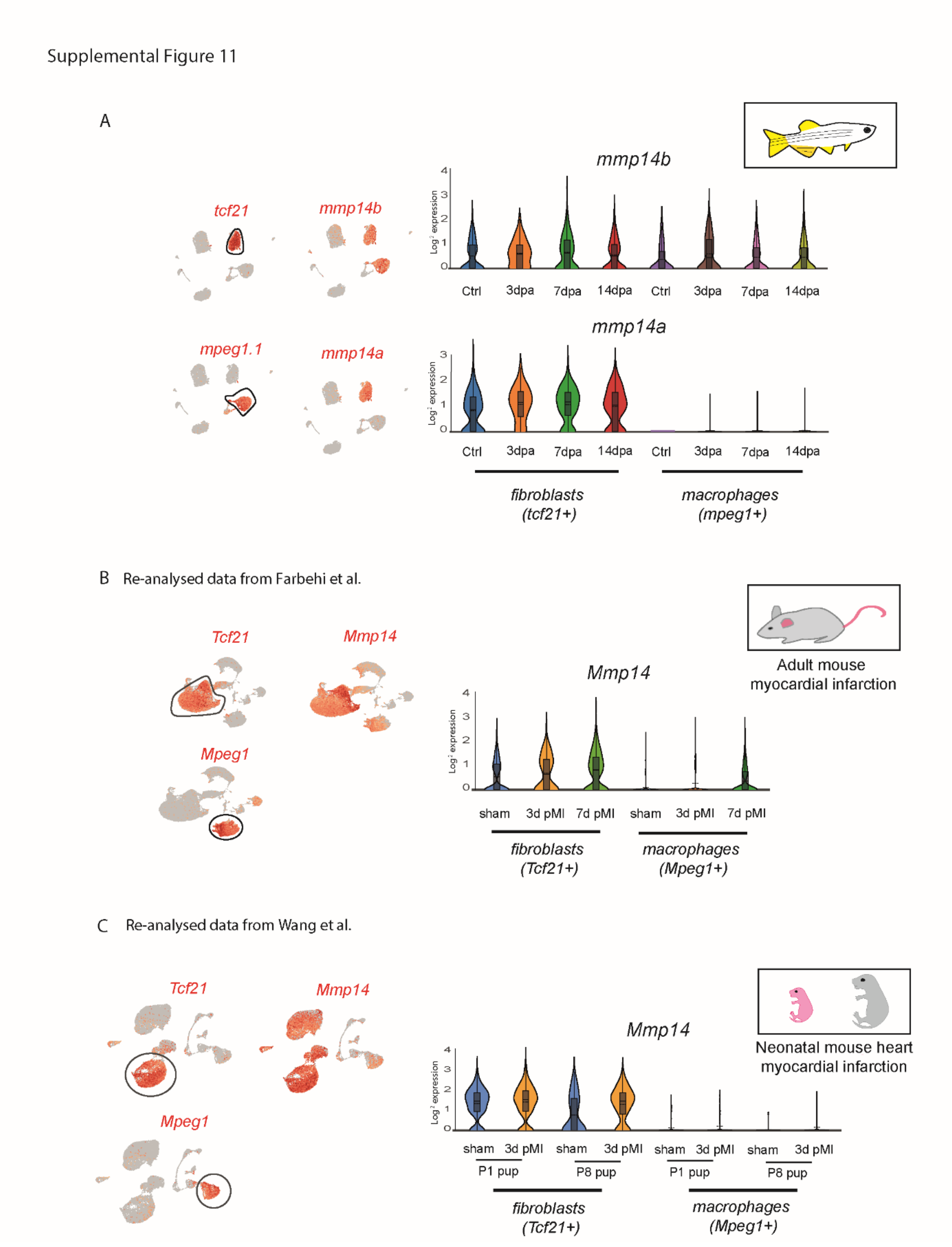

Supplemental figure S11. Comparison of Mmp14 expression in macrophages between species. (A) Adult zebrafish apical resection. UMAP plots depicting the expression of *mmp14b* and *mmp14a* in relation to *tcf21* expressing fibroblasts and *mpeg1.1* expressing macrophages. Note *mmp14b* is

expressed by fibroblasts and macrophages while *mmp14a* is restricted to fibroblasts. Violin plots showing the expression of *mmp14b* and *mmp14a* at different time points during regeneration (uninjured (ctrl), 3dpa, 7dpa and 14dpa) in fibroblasts and macrophages. (**B**) Adult mouse myocardial infarction. Re-analysed UMAP plots depicting the expression of *Mmp14* in relation to *Tcf21* expressing fibroblasts and *Mpeg1* expressing macrophages. Violin plots showing the expression of *Mmp14* at different time points after injury (sham, 3 days post myocardial infarction (3d pMI) and 7d pMI) in fibroblasts and macrophages. Note that *Mmp14* expression appears at 7d pMI in the macrophage population. (**C**) Neonatal mouse myocardial infarction. Re-analysed UMAP plots depicting the expression of *Mmp14* in relation to *Tcf21* expressing fibroblasts and *Mpeg1* expressing macrophages. Violin plots showing the expression of *Mmp14* at different time points after injury (sham, 3d pMI) by fibroblasts and macrophages in regenerating P1 pups and non-regenerating P8 pups. Note that *Mmp14* expression is absent in the macrophage population.

Supplemental Figure 12

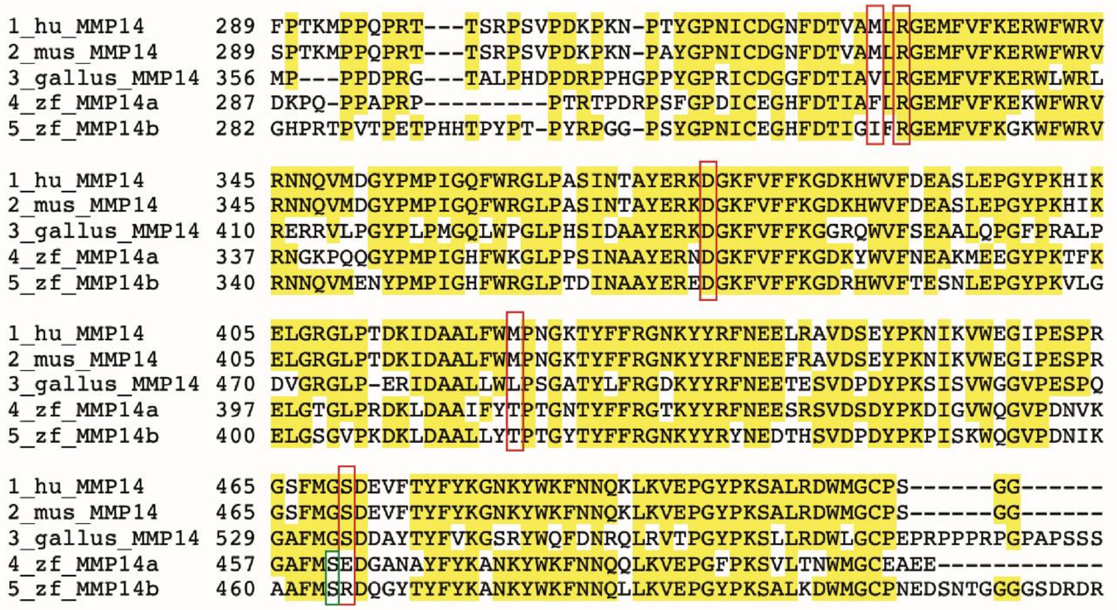

Supplemental figure S12. Homology of MMP14 between species. Alignments of MMP14 from human (hu_MMP14), mouse (mus_MMP14), chicken (gallus_MMP14) and zebrafish (zf_MMP14a and zf_MMP14b). The red boxes denote the amino acids (AA) which are predicted to be in proximity to NSC405020 within the PEX domain of MMP14 in human and mouse. Of the 5 AA's predicted to be in proximity to NSC405020, 3 of these are also conserved with chicken (R330, D376 and S478). Of these 3 AA's 2 are conserved in zebrafish ((R330 and D376) while the third AA (S478) is shifted 1 residue towards the N-term (highlighted with a green box) in zebrafish.

Supplemental Figure 13

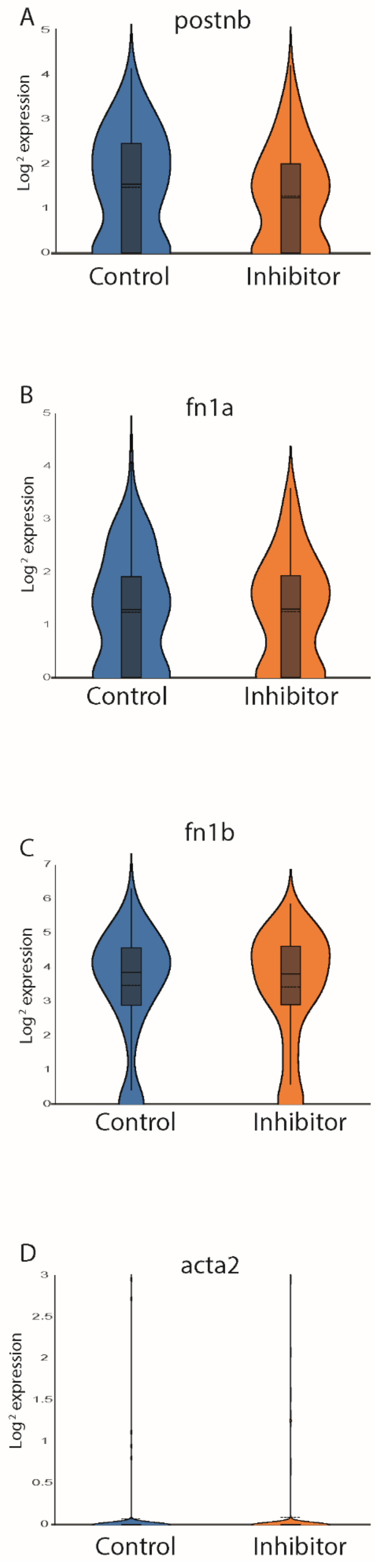

Supplemental figure S13. Comparison of gene expression by fibroblasts following Mmp14 inhibition. (**A-D**) Violin plots comparing the expression of fibrosis associated genes within the fibroblast population in untreated (Control) or MMP14 inhibitor treated (Inhibitor) regenerating zebrafish hearts, *postnb* (**A**), *fn1a* (**B**), *fn1b* (**C**) and *acta2* (**D**).

Supplemental Figure 14

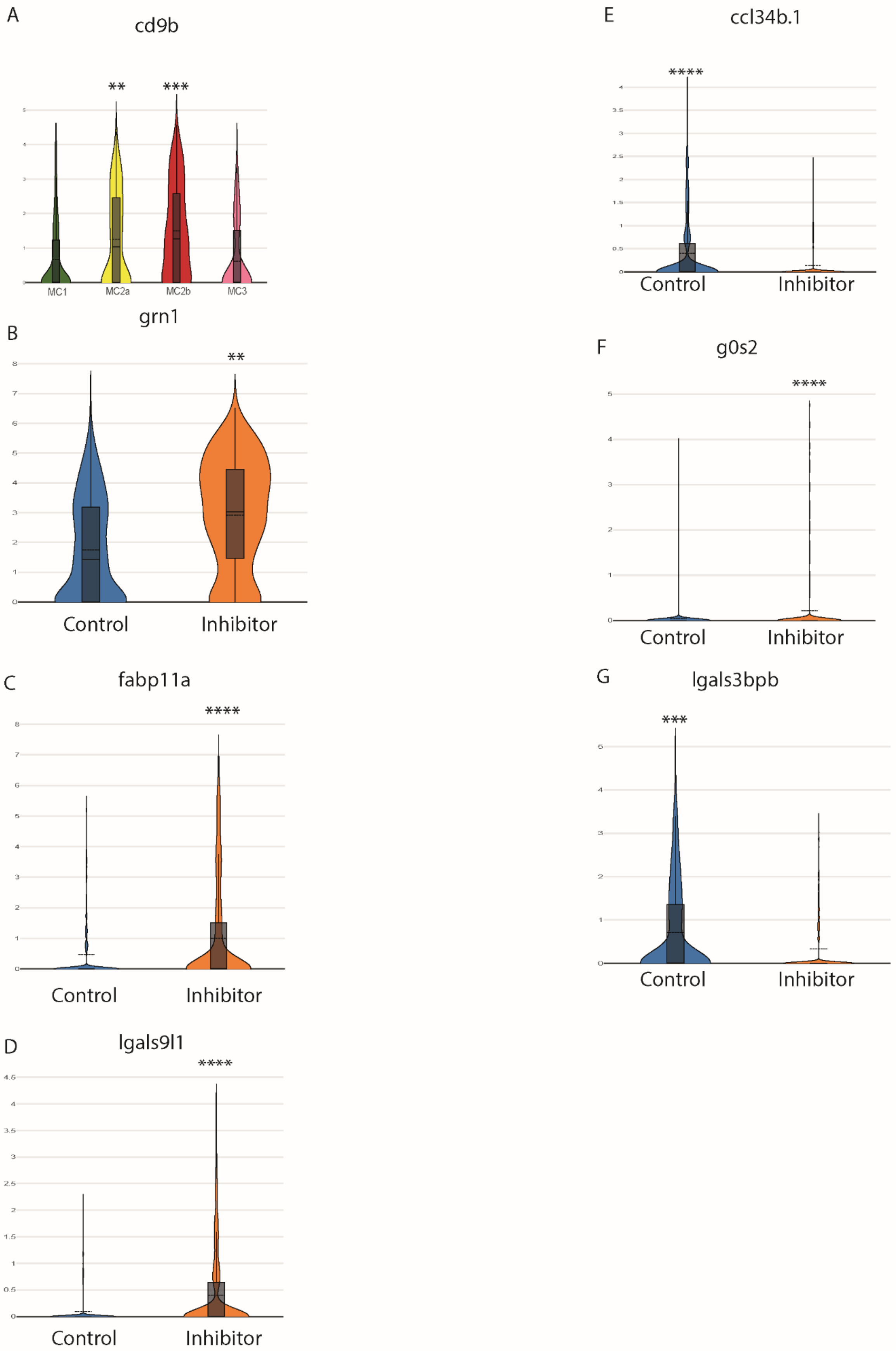

Supplemental figure S14. Comparison of gene expression within macrophages following Mmp14 inhibition. (**A**) Violin plot indicating the expression of *cd9b* within the different macrophage subpopulations (MC1, MC2a, MC2b and MC3). (**B-G**) Violin plots comparing the expression of regeneration associated genes within the macrophage population in untreated (Control) or MMP14 inhibitor treated (Inhibitor) 3dpa regenerating zebrafish hearts, *grn1* (**B**), *fabp11a* (**C**), *lgals9l1* (**D**), *ccl34b.1* (**E**), *g0s2* (**F**) and *lgals3bpb* (**G**). *P* values were adjusted using the Benjamini-Hochberg correction. *$P<0.1$, **$P<0.05$, ***$P<0.01$, **** $P<0.001$.

Supplemental file legends

Supplemental File S1. Table showing significant differentially expressed genes in each sub-cluster of cells.

Supplemental File S2. Table showing significant differentially expressed genes in each cell type.

Supplemental File S3. Table indicating the number of hearts per condition, the number of cells sequenced per condition and the median genes per cell.

Supplemental Materials and Methods

Single cell RNA sequencing.

Hearts were dissected, placed in cold HBSS with heparin. Atria and outflow tracts removed, ventricles opened and washed. 10 ventricles were pooled for each sample: unamputated, sham-operated, 3 dpa, 3 dpa +vehicle, 3 dpa+inhibitor, 7 dpa and 14 dpa. Cells were pre-incubated for 2h on ice in HBSS-7% TrypLE. HBSS TrypLE was removed and tissue was dissociated in HBSS Collagenase (type II, 5mg/ml; type IV, 5mg/ml) with CaCl2 (12µM) for 1h on a rotator (800RPM) at 32 degree. Dissociated hearts were put through a 40µm cell strainer, spun down (300g, 5min at 4 degrees) and recovered in FACS buffer (HBSS 2% FCS). Nuclei were stained by adding Vybrant DyeCycle Ruby Stain to the cell suspension and heated for 10min at 32 degrees before adding DAPI and sorting into PBS 0.04% BSA. Sorting was performed on a BD FACS Melody.

Cellular suspensions were loaded on a Chromium controller (10x Genomics, Pleasanton, CA, USA) to generate single-cell Gel Beads-in-Emulsion (GEMs). Single-cell RNA-Seq libraries were prepared using Chromium Single cell 3'RNA Gel Bead and Library Kit v3.1. GEM-RT was performed in a C1000 Touch Thermal cycler with 96-Deep Well Reaction Module (Bio-Rad): 53°C for 45 min, 85°C for 5 min; held at 4°C. After RT, GEMs were broken and the single-strand cDNA was cleaned up with DynaBeads MyOne Silane Beads (Thermo Fisher Scientific). cDNA was amplified using the C1000 Touch Thermal cycler with 96-DeepWell Reaction Module: 98°C for 3 min; cycled 12: 98°C for 15 s, 63°C for 20 s, and 72°C for 1 min; 72°C for 1 min; held at 4°C. Amplified cDNA product was cleaned up with the SPRI select beads. Indexed sequencing libraries were constructed following these steps: (1) fragmentation, end-repair and A-tailing and size selection with SPRIselect; (2) adapter ligation and cleanup with SPRIselect; (3) sample index PCR and size selection with SPRIselect. The barcoded sequencing libraries were quantified by quantitative PCR (KAPA Biosystems Library Quantification Kit for Illumina platforms). Sequencing libraries were loaded at 300 pM on an Illumina NovaSeq6000

using the following read length: 28 bp Read1, 8 bp I7 Index, 91 bp Read2 (experiment 1), and 28 bp Read1, 10 bp I7 Index, 10 bp I5 Index, 87 bp Read2 (experiment 2).

Image analyses and base calling were performed using the NovaSeq Control Software and the Real-Time Analysis component (Illumina). Demultiplexing was performed using the 10X Genomics software Cellranger mkfastq (v3.1.0 for experiment 1 and v6.0.1 for experiment 2), a wrapper of Illumina's bcl2fastq (v2.20). The quality of the raw data was assessed using FastQC (v0.11.8) from the Babraham Institute and the Illumina software SAV (Sequencing Analysis Viewer). FastqScreen (v0.14.0) was used to estimate the potential level of contamination.

Alignment, gene expression quantification and statistical analysis were performed using Cell Ranger count on *Danio rerio* 's transcriptome GRCz11 (sequences and annotation were downloaded from Ensembl! on July 24th, 2019). In order to discard ambient RNA falsely identified as cells, Cell Ranger count was run a second time with the option --force-cells to force the number of cells to detect. Cell Ranger aggr was then used to combine each sample result into one single analysis.

Data availability

Datasets generated for this study: sequencing data have been deposited in the ArrayExpress database at EMBL-EBI (www.ebi.ac.uk/arrayexpress) under accession code E-MTAB-10643.

Previously published datasets used for this study: E-MTAB-7376 (www.ebi.ac.uk/arrayexpress) from Farbehi et al.(Farbehi, Patrick et al. 2019); GSE153480 (www.ncbi.nlm.nih.gov/geo/) from Wang et al.(Wang, Cui et al. 2020).

Edu labelling

EdU labelling was performed according to the manufacturers instructions (Click-iT EdU Kit C10337, Molecular Probes). Amputated adult *Tg(mpeg1.1:mCherry^{+})* or ABWT were anesthetized in Tricaine

then injected with 50μl of a 240μg/ml EdU solution daily. Following processing and imaging EdU labelled cardiomyocytes were counted using IMARIS software.

Immunohistochemistry

Immunohistochemistry was performed on 10 μm cryo-sections as previously described(Lai, Marin-Juez et al. 2017). The primary antibodies used in this manuscript are anti-RFP (5F8 Chromotek), anti-GFP (GFP1020 Aves), anti-Acta2 GTX124505 Genetex), anti-RSPO1 (ab106556 Abcam), anti-Tal11 (abx339062 Abbexa), anti-α Sarcomeric actin (A2172 Sigma), anti-MMP14 (mbs422986 Mybiosource and GTX128198 Genetex), anti-MEF2c (ab197070), IB4 (Isolectin GS1B4) (I21413 Invitrogen), EdU labelling was performed according to the manufacturers instructions (Click-iT EdU Kit C10337, Molecular Probes).

Cell counting

For determining the proportions of different cell types, hearts were processed for cryosectioning. 5 12-14μm cryosections were stained and 8-10μm z-stacks spanning the ventricle, as described in Figure ure 1F, were acquired (Axioplan2, ApoTome system, Zeiss, Germany). Individual nuclei were manually identified (Imaris), and assigned based on their association with lineage markers. For quantification of αSMA^{+} and Col1a2:mCherry^{+} cells, 5 12-14μm cryosections were stained and 8-10μm z-stacks spanning the apex were acquired, and cells quantified as described above.

Histology

Acid Fuchsin-Orange G (AFOG) staining was performed on 10 μm cryosections as previously described (Poss, Wilson et al. 2002). The area of the wound was calculated using ImageJ software.

Macrophage counting

IB4 labelled macrophages were counted using IMARIS software. For proximal macrophages we counted macrophages which were within 100μm of the GFP labelled myocardium. For distal macrophages we counted macrophages which were within 100μm of the edge of the scar/wound region.

Imaging

A Zeiss Discovery V20 fluorescence stereomicroscope fitted with a Tucsen FL20 Microscope Camera was used for histological section imaging and either a Zeiss Axio Imager equipped with an Apotome 3 module or a Leica TCS SP-8 confocal microscope were used for imaging IHC labeled sections. Image analysis was performed using IMARIS software.

Dominant negative Tal transgenic zebrafish

The DN *tal* construct and transgenic line were generated using the Tol2 Kit as described (Kwan, Fujimoto et al. 2007, Jopling, Sleep et al. 2010). Dominant negative zf *tal1* was generated as described(Aplan, Nakahara et al. 1992). For the *Tg(fliEP:loxRFPlox:DNtal*) construct the 5´ entry clone 478 p5Efli1ep was a gift from Nathan Lawson (Addgene plasmid # 31160 ; http://n2t.net/addgene:31160 ; RRID:Addgene_31160, the middle entry clone contained a floxed RFP stop cassette amplified from pBOB-LRL-CBReGFPpA (a kind gift from Geoff Whal) and the 3´ entry clone contained zebrafish dominant negative *tal1*. For the *Tg*(*fliEP:Ert2CreErt2)* the 5´ entry clone was p5Efli1ep and the middle entry clone was pMEErt2CreErt2 as described(Jopling, Sleep et al. 2010).

Real-time quantitative RT-PCR

RNA was extracted from amputated/unamputated ventricles of AB wild type zebrafish and quantitative PCR was performed using a Roche LightCycler 480 system as described (Jopling, Sune et al. 2012). Primer sequences are provided below.

MMP2 forward- 5’ GGTGTGCAACCACTGAAGAT 3’

MMP2 reverse- 5’ AGGGTGCTCCATCTGAATTT 3’

MMP9 forward- 5’ TTTGACGCCATCACTGAAAT 3’

MMP9 reverse- 5’ TTCGCAGAGATCATGAAAGG 3’

MMP13a forward- 5’ CTCAGAGCCCAGATGTTGAA 3’

MMP13a reverse- 5’ CCTTCTCACCTTTGATCAGGA 3’

MMP14a forward- 5’ CTCGCAAGTGTGTTTCTGGT 3’

MMP14a reverse- 5’ TCACCAGGAGGAAGATACCC 3’

MMP14b forward- 5’ GATATGAAACCTGAGGCATGG 3’

MMP14b reverse- 5’ GTGACTGTCAGGCCGTAGAA 3’

Chemical treatments

Transgenes were expressed by inducing Cre mediated recombination with tamoxifen as described (Jopling, Sleep et al. 2010). GM6001 (ab120845, Abcam) was dissolved in DMSO to reach a stock concentration of 10 mM and then split into 10 μl aliquots and stored at – 20°C. On the day of injection, the 10 μl aliquot was added to 1 ml 1X PBS to reach a final concentration of 100 μM. This solution was administered daily by interperitoneal injection. Control groups were administered daily with 1X PBS. NSC405020 (SML0518 Sigma) was diluted in DMSO to reach a stock concentration of 10 mM. 5 fish were placed in a beaker with 500ml of system water and 500μl of NSC405020 was added to reach a final concentration of 10 μM. Controls were placed in a beaker and 500μl of DMSO was added. The fish were left overnight then rinsed the next morning and returned back to the main aquarium during the day. This was repeated for the duration of each experiment. All chemical treatments were performed in accordance with local approval (APAFIS#4054).

Neonatal heart regeneration

All amputations were performed on Swiss-JL (Janvier labs) P1 neonatal mice as described(Mahmoud, Porrello et al. 2014),in accordance with local approval (APAFIS#1498-15516).

Statistical analysis

GraphPad prism was used to perform all statistical analysis. Details of statistical analysis are provided in Figures and Figure legends.

References

Aplan, P. D., K. Nakahara, S. H. Orkin and I. R. Kirsch (1992). "The SCL gene product: a positive regulator of erythroid differentiation." EMBO J **11**(11): 4073-4081.

Farbehi, N., R. Patrick, A. Dorison, M. Xaymardan, V. Janbandhu, K. Wystub-Lis, J. W. Ho, R. E. Nordon and R. P. Harvey (2019). "Single-cell expression profiling reveals dynamic flux of cardiac stromal, vascular and immune cells in health and injury." Elife **8**.

Jopling, C., E. Sleep, M. Raya, M. Marti, A. Raya and J. C. Belmonte (2010). "Zebrafish heart regeneration occurs by cardiomyocyte dedifferentiation and proliferation." Nature **464**(7288): 606-609.

Jopling, C., E. Sleep, M. Raya, M. Marti, A. Raya and J. C. Izpisua Belmonte (2010). "Zebrafish heart regeneration occurs by cardiomyocyte dedifferentiation and proliferation." Nature **464**(7288): 606-609.

Jopling, C., G. Sune, A. Faucherre, C. Fabregat and J. C. Izpisua Belmonte (2012). "Hypoxia induces myocardial regeneration in zebrafish." Circulation **126**(25): 3017-3027.

Kwan, K. M., E. Fujimoto, C. Grabher, B. D. Mangum, M. E. Hardy, D. S. Campbell, J. M. Parant, H. J. Yost, J. P. Kanki and C. B. Chien (2007). "The Tol2kit: a multisite gateway-based construction kit for Tol2 transposon transgenesis constructs." Dev Dyn **236**(11): 3088-3099.

Lai, S. L., R. Marin-Juez, P. L. Moura, C. Kuenne, J. K. H. Lai, A. T. Tsedeke, S. Guenther, M. Looso and D. Y. Stainier (2017). "Reciprocal analyses in zebrafish and medaka reveal that harnessing the immune response promotes cardiac regeneration." Elife **6**.

Mahmoud, A. I., E. R. Porrello, W. Kimura, E. N. Olson and H. A. Sadek (2014). "Surgical models for cardiac regeneration in neonatal mice." Nat Protoc **9**(2): 305-311.

Poss, K. D., L. G. Wilson and M. T. Keating (2002). "Heart regeneration in zebrafish." Science **298**(5601): 2188-2190.

Wang, Z., M. Cui, A. M. Shah, W. Tan, N. Liu, R. Bassel-Duby and E. N. Olson (2020). "Cell-Type-Specific Gene Regulatory Networks Underlying Murine Neonatal Heart Regeneration at Single-Cell Resolution." Cell Rep **33**(10): 108472.

Supplemental file 3

**Sample**	**unamputated control**	**3 days post-amputation**	**7 days post-amputation**	**14 days post-amputation**
number of hearts pooled/dissociated	10	10	10	10
number of cells	1 876	1 565	3 153	2 475 cells
median genes per cell	1 411	1 339	1 425	1 450
